# Single-cell analysis defines highly specific leukemia-induced neutrophils and links MMP8 expression to recruitment of tumor associated neutrophils during FGFR1 driven leukemogenesis

**DOI:** 10.1186/s40164-024-00514-6

**Published:** 2024-05-10

**Authors:** Tianxiang Hu, Bo Cheng, Atsuko Matsunaga, Ting Zhang, Xiaocui Lu, Hui Fang, Stephanie F. Mori, Xuexiu Fang, Gavin Wang, Hongyan Xu, Huidong Shi, John K. Cowell

**Affiliations:** 1 Georgia Cancer Center, 1410 Laney Walker Blvd, 30912 Augusta, GA USA; 2 Department of Stomatology, Zhongnan Hospital of Wuhan University, Wuhan, China; 3 Department of Dermatology, Tianjin Academy of Traditional Chinese Medicine Affiliated Hospital, Tianjin, China; 4 University of Georgia, Athens, GA USA; 5 Department of Biostatistics, Data Science and Epidemiology, School of Public Health, Augusta University, 30912 Augusta, GA USA

**Keywords:** Leukemia, FGFR1, TME, PMN-MDSC, TAN, scRNA-Seq, MMP

## Abstract

**Background:**

Leukemias driven by activated, chimeric FGFR1 kinases typically progress to AML which have poor prognosis. Mouse models of this syndrome allow detailed analysis of cellular and molecular changes occurring during leukemogenesis. We have used these models to determine the effects of leukemia development on the immune cell composition in the leukemia microenvironment during leukemia development and progression.

**Methods:**

Single cell RNA sequencing (scRNA-Seq) was used to characterize leukemia associated neutrophils and define gene expression changes in these cells during leukemia progression.

**Results:**

scRNA-Seq revealed six distinct subgroups of neutrophils based on their specific differential gene expression. In response to leukemia development, there is a dramatic increase in only two of the neutrophil subgroups. These two subgroups show specific gene expression signatures consistent with neutrophil precursors which give rise to immature polymorphonuclear myeloid-derived suppressor cells (PMN-MDSCs). Analysis of gene expression in these precursor cells identified pathways that were specifically upregulated, the most pronounced of which involved matrix metalloproteinases *Mmp8* and *Mmp9*, during leukemia progression. Pharmacological inhibition of MMPs using Ilomastat preferentially restricted in vitro migration of neutrophils from leukemic mice and led to a significantly improved survival in vivo, accompanied by impaired PMN-MDSC recruitment. As a result, levels of T-cells were proportionally increased. In clinically annotated TCGA databases, *MMP8* was shown to act as an independent indicator for poor prognosis and correlated with higher neutrophil infiltration and poor pan-cancer prognosis.

**Conclusion:**

We have defined specific leukemia responsive neutrophil subgroups based on their unique gene expression profile, which appear to be the precursors of neutrophils specifically associated with leukemia progression. An important event during development of these neutrophils is upregulation *MMP* genes which facilitated mobilization of these precursors from the BM in response to cancer progression, suggesting a possible therapeutic approach to suppress the development of immune tolerance.

**Supplementary Information:**

The online version contains supplementary material available at 10.1186/s40164-024-00514-6.

## Background

Hematologic malignancies associated with fibroblast growth factor receptor (FGFR1) abnormalities present in heterogeneous forms, including myeloproliferative neoplasm, acute myeloid leukemia (AML), T- or B-lineage lymphoblastic leukemia/lymphoma, and even mixed phenotype acute leukemia [1]. The hallmark of this stem cell leukemia/lymphoma (SCLL) syndrome is the presence of chimeric kinases formed as a result of chromosome translocations [2], which lead to a ligand-independent, constitutive activation of FGFR1 signaling [3]. Bone marrow (BM) transduction and transplantation with FGFR1 fusion kinases can independently transform normal mouse [4–6] and human [7, 8] hematopoietic stem cells into leukemia cells, and suppression of FGFR1 activation using kinase inhibitors [9–11] can suppress leukemogenesis in humanized and syngeneic animal models, suggesting that this is the critical driver event in the disease. The homogenous genetic background in SCLL provides an ideal model for studying mechanisms underlying leukemogenesis, including immune evasion.

The immune system plays a critical role in preventing tumor growth and expansion through processing and presentation of tumor specific antigens and removal of tumor by activated effector T cells [12–15]. However, despite this immune surveillance, tumors still manage to develop in the presence of a functioning immune system, through the development of immune escape mechanisms leading to tolerance [16, 17]. Part of this process involves expansion of immature myeloid cells referred to as myeloid derived suppressor cells (MDSCs), which are characterized functionally by their ability to suppress immune responses [18–20].

To date much of our understanding of the cancer-related mechanisms behind MDSC-induced immune escape has centered on studies of solid tumors [21, 22]. Very little, however, is known about the immune escape process involved in the development of leukemia which, since they have a very different pathological presentation compared with solid tumors, are likely to involve leukemia-specific mechanisms. To address this issue, we have performed extensive investigation using our well-established animal models of the SCLL syndrome, which are characterized by the constitutive activation of chimeric FGFR1 kinases [4–6]. In a recent study we showed that engrafted SCLL cells suppressed the anti-leukemia immune response through induction of MDSC and subsequent suppression of CD4 + and CD8 + cells, to ensure leukemia development [23]. In mice, there are two types of MDSC, monocytic (M)-MDSC and polymorphonuclear (PMN)-MDSC [24–26]. Murine MDSC express CD11b and the M-MDSC also express high levels of Ly6C, while the PMN-MDSC express Ly6G in addition to intermediate levels of Ly6C. PMN-MDSCs and tumor-associated neutrophils (TANs) share both the origin and many morphological and phenotypic features, and overlap with each other especially in a pro-tumor context [21, 27, 28].

Using scRNA-Seq, we have demonstrated a global suppression of immune effector cells by MDSCs. Further characterization of PMN-MDSCs/neutrophils identified two leukemia-induced neutrophil subtypes with unique gene expression profiles during leukemia progression. Significantly, *Mmp 8 and 9* were upregulated and pharmacological inhibition of MMPs leads to reduced mobilization of PMN-MDSC precursors from the BM into the peripheral blood (PB) with a concomitant increase in T-cells. As a result, survival of the drug-treated mice is improved significantly. The clinical significance of *MMP8/9* was further analyzed in TCGA datasets [29–32], revealing significant correlations between expression levels, neutrophil infiltration and cancer survival in AML and other tumors.

## Methods

### Mouse engraftment and flow cytometry analysis

0.25 × 10^6^ SCLL cells were engrafted into 6–8-week-old, female Balb/C mice and PB was collected for flow cytometry analysis as described previously [23]. All animal experiments were performed under an approved protocol from the Augusta University Institutional Animal Care and Use Committee. Flow antibodies used in this study include CD4-APC (Biolegend, #100,412), CD8α-PE/Cy7 (Biolegend, #100,722), Ly6C-PE (Biolegend, #128,008), CD11b-PerCP/Cy5.5 (Biolegend, #101,228), Ly6G-APC/Cy7 (Biolegend, #127,624), CD19-Violet 421 (Biolegend, #115,538), CD49b-PerCP/Cy5.5 (Biolegend, #108,916).

### Single-cell RNA sequencing (scRNA-seq) and analysis

PB samples were depleted of red cells, and dead cells were removed using the Miltenyi dead cell removed kit (#130-090-101), whereafter viable leukocytes were processed for scRNA-Seq libraries using the Chromium Controller (10X Genomics) and the Chromium 3′ Single Cell mRNA-seq V3 reagents. The scRNA-Seq libraries were sequenced using an Illumina NovaSeq 6000 System. The raw data were processed using the Cell Ranger package (10X Genomics) and outputs were analyzed using Seurat v4.3 [33]. Quality control measures were implemented in Seurat to filter out cells expressing a high number of genes (top 2 percentile, assuming a 2% doublet rate) or with a higher percentage of mitochondrial genes (percentage of mt > 10) or hemoglobin (percentage of Hbb > 2.5) genes. Hemoglobin genes used for calculation include “Hba-a1”, “Hba-a2”, “Hbb-bh1”,“Hbb-bh2”, “Hbb-bs”, “Hbb-bt”, “Hbb-y”. Additional QC included removing UMIs with a number of features less than 200 (low read counts). We used the standard Seurat pipeline running NormalizeData, FindVariableFeatures (selection.method =”vst”, nfeatures = 2000), and ScaleData. The doublets were further removed with the ‘DoubletFinder’ package V3. Datasets were then integrated using the canonical correlation analysis (CCA)-based integration approach in Seurat to remove batch effects. Anchor genes were identified using the Seurat FindIntegrationAnchors function after normalization. The IntegrateData function merged the datasets. Subsequently UMAP and tSNE clustering was performed in the integrated assay using the anchor genes. RunPCA (npcs = 30), RunUMAP (dim = 1:30), RunTSNE (dim = 1:30), FindNeighbours (dims = 1:30), and FindClusters (resolution = 1) was used to first visualize the data, which revealed 20 clusters. Differential expression analysis was performed using the Seurat FindMarkers or FindAllMarkers functions using log_2_ normalized and scaled data before integration, with the threshold of min.pct = 0.25 and logfc.threshold = 0.25. Cell type was assigned to each cluster using the cluster identity predictor (CIPR) algorithm in combination with manual correction based on the most highly expressed markers identified in each cluster (Supplemental Table 1).

For a focused analysis of neutrophil populations, all neutrophil clusters were subgrouped and then the same clustering and marker identification processes were performed. In addition, the differential gene and pathway analysis was performed with indicated neutrophil clusters at different disease stages. The single-cell trajectory and pseudotime analysis was also performed in the neutrophil clusters to predict potential differentiation processes among clusters.

### Pathway and gene signature enrichment analysis

The pathway enrichment analysis of differentially expressed genes between clusters or groups was performed using the clusterProfiler v4.0 R package [34]. The single-cell level pathway analysis was performed using the AUCell v1.16.0 R package [35]. Pathway gene sets were downloaded from the WikiPathway collection using the version of “wikipathways-20190510-gmt-Mus_musculus.gmt” and used to calculate the pathway activity score, indicating whether a critical subset of the input gene set is enriched within the expressed genes for each cell (Supplemental Table 2). The AUC score matrix was imported into Seurat to generate violin, tSNE, and UMAP plots. Heatmaps of gene expression levels in single cells were generated using the ComplexHeatmap package.

### Single-cell trajectory and pseudotime analysis

The pseudotemporal and trajectory analysis was performed using Monocle 3 with default settings. The Seurat object with cluster IDs was exported as CellDataSets and directly imported into Monocle3 v1.3.1 [36]. The marker genes identified by the Seurat FindAllMarkers function were used in ordering cells along the pseudotime trajectory. The DDRTree method was used for dimension reduction and trajectory inference. Branch analysis was performed using the Monocle2 Branched Expression Analysis Modeling (BEAM) function. One hundred thirty genes (adjusted *P* < 0.001) with expression patterns that are pseudotime dependent were used to plot heatmaps (K means = 6). Six gene blocks were chosen according to the distinct patterns of gene expression change toward the pseudotime.

### MMP inhibitor treatment in vitro and in vivo

The broad-spectrum matrix metalloprotease (MMP) inhibitor, Ilomastat (Synonyms: GM6001; Galardin) was purchased from MedChemExpress. For in vitro transwell migration assays, magnetically isolated Ly6G + neutrophils were treated with 50µM Ilomastat at 1 × 10^6^ cell/ml in 6 well plates for 8 h, and then 0.1 × 10^6^ pretreated cells were seeded into 3 μm pore transwell chambers (Corning, CLS3462) for a further 24 h. The non-migrated cells were removed using a cotton swab, and migrated cells were first fixed and then stained with Crystal Violet for imaging and quantification analysis. Migration was calculated as described previously [37]. For in vivo treatment, Ilomastat was administrated IP on 7 consecutive days at 100 mg/kg body weight starting 7 days after engraftment [38, 39]. Immune monitoring and leukemia progression were achieved as described [23].

### Correlative analysis in cancer patient samples

For correlative analysis of *MMP8* and *9* in AML patients, the transcriptomic sequencing of samples from the Beat AML program were used [30]. The whole exome sequencing data for Pediatric Acute Lymphoid Leukemia (ALL) in Phase II clinical trial (TARGET, 2018) were used for correlative analysis in ALL [29]. Analyses were performed directly using cBioPortal [31, 32], with the comparison between the top and bottom quartiles. Further validation of the AML correlation was performed in an independent AML dataset using the R2 platform [40]. For multivariate analysis, the AML data set was downloaded from cBioPortal. We performed multivariate survival analysis using the Cox proportional hazard model. In addition to the molecular markers, we included additional patient-level covariates such as age, sex, stage/relapse in the model for KIRC and AML patients [29, 30]. The analysis was performed in R 4.3.0. For analysis of the association between neutrophil infiltration and gene expression, the TIMER2.0 online platform was used [41]. The correlation between gene expression level and purity or infiltration levels were exemplified in READ and THCA data set for *MMP8*, and the COAC and THCA data set for *MMP9*. Correlation analysis between cancer patient survival and target gene expression, mutation, methylation and copy number alterations (CNAs) was performed using the online TCGA-survival platform [42].

## Results

### Immune suppression by emerging MDSC during leukemia progression

**Fig. 1:**
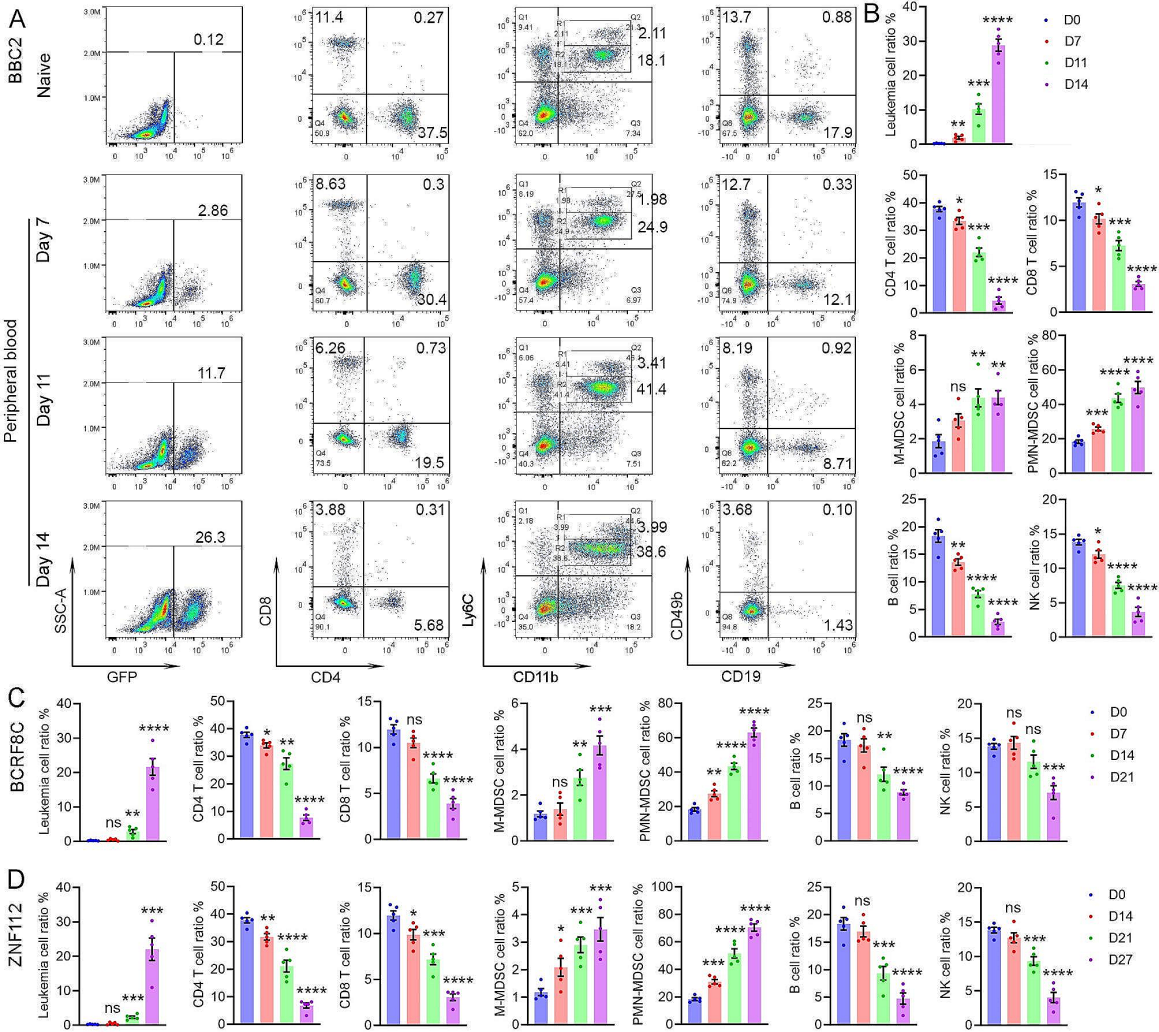
Flow cytometry monitoring of the major immune cells during SCLL development. Representative flow diagrams of peripheral blood (PB) samples from naive mice (**A**) give the baseline levels of CD4 + and CD8+, T-cells, Ly6C + CD11b + myeloid cells as well as CD19 + B-cells and CD49b + NK cells. When the same markers are analysed in PB samples from mice which have been engrafted with BBC2 SCLL cells, there is a progressive decrease in the number of T-, B- and NK cells over 7 days (D7-D14) and a progressive increase in the number of Ly6C + CD11b + cells. While the Ly6C^hi^CD11b + cell population (M-MDSC) shows only a modest increase, the proportion of Ly6C^int^D11b + PMN-MDSC show a highly significant increase. Leukemic cells are defined by the expression of GFP. The mean of the percentage changes in the various cell types across all five mice in each cohort (indicated by the colored dots) defines consistent changes (**B**). When a similar analysis is performed in mice engrafted with AML-like BCRF8C SCLL cells (**C**) or T-cell leukemic ZNF112 SCLL cells (**D**), the same distribution of the different immune cells across the time scale is consistently seen. In all cases a two-way comparison between the naïve and leukemic mice was performed using the student’s t-test providing the relative significances; ns = not significant, * *p* < 0.01, ** *p* < 0,001, *** *p* = 0.0001, **** *p* = < 0.00001. The longer time periods of sampling in (**C**) and (**D**) reflects a slower progression of disease in these models

To assess the impact of leukemic cells on the composition of the microenvironment, PB samples were obtained from GFP + BBC2 cell engrafted mice. The BBC2 cell line was derived from a primary murine SCLL leukemia [5] and expresses the BCR-FGFR1 chimeric kinase. BBC2 cells have a pro-B cell immunophenotype, although with mixed linage markers [5]. When engrafted into syngeneic hosts, leukemia develops within 16 days, which provides a rapid model to detect genetic and phenotypic changes associated with leukemogenesis. PB samples were collected at days 7 (early-stage), 11 (mid-stage) and 14 (late-stage) after engraftment to monitor dynamic changes of immune cell composition during leukemic progression. Representative flow cytometric analysis at these time points for CD4+/ CD8 + T-cells and Ly6C+/CD11b + myeloid cells are shown in Fig. 1A. GFP + leukemia in this model appears in PB at day 7 and becomes maximal in the D14 cohort. Levels of Ly6C^hi^CD11b + cells, representing the M-MDSC, represent only a small population in naive mice but increase proportionally with the increase in tumor cells over time. The Ly6C^int^CD11b + PMN-MDSC in the tumor bearing mice, however, become the predominant population. Analysis of CD19 + B-cells and CD49b + NK cells over the same time course shows a progressive reduction in population levels (Fig. 1A). These changes were consistent across all five mice in each cohort (Fig. 1B).

To extend these studies to other SCLL models, we performed the same flow cytometric analysis in mice engrafted with the AML-like BCRF8C cells [43] expressing BCR-FGFR1 (Fig. 1C) and ZNF112 T-cell leukemia cells [4] expressing the ZMYM2-FGFR1 chimeric kinase (Fig. 1D). Leukemogenesis in these models develops more slowly than for BBC2 cells. PB sampling for TME analysis, therefore, was taken at later times. BCRF8C and ZNF112 engrafted mice succumbed from leukemia after approximately 22 and 28 days, respectively, with relative lower levels of GFP + leukemia cells in the PB compared to ∼ 15 days for BBC2 cells. In both cases the similar and continuous induction of MDSCs and consequential suppression of immune effector cells, including T, B and NK cells observed for BBC2 cells were also evident in these mice (Fig. 1C, D and Supplemental Fig. 1). These observations of increased MDSCs and decreased immune effector cells were further confirmed by absolute cell counts (Supplemental Fig. 1C and D). Noticeably, ZNF112 induced more PMN-MDSCs compared with the BBC2 and BCRF8C models, which show higher induction of CD11b + Ly6C^low^ myeloid cells (Fig. 1D and Supplemental Fig. 1A). These observations demonstrate the consistency of the immune suppression by MDSCs across independently derived SCLL cells.

### Single cell profiling of the dynamic immune cell composition during leukemogenesis

**Fig. 2:**
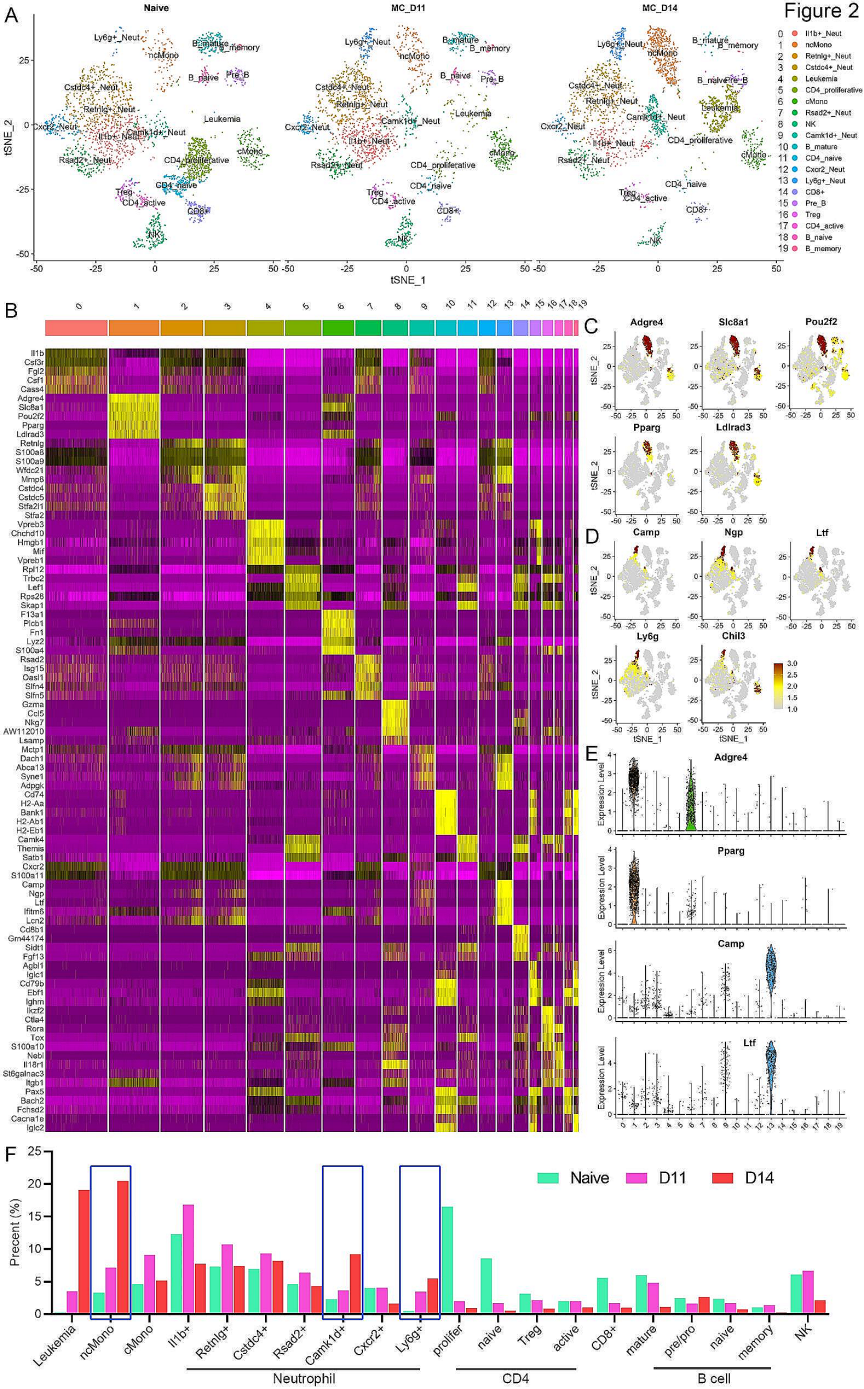
Single cell profiling of the immune cell composition during SCLL progression. tSNE distribution of cells in the PB from naive and BBC2 (D11 and D14) engrafted mice (**A**) identified 20 distinct clusters, which are annotated based on their specific gene expression profiles as shown in the heatmap in (**B**), the 5 most statistically significant genes as defined by the FindAllMarkers function. Feature plots for selected genes relative to the tSNE profile shown in (**A**), clearly show their predominant or specific expression in the non-classical monocyte cluster (**C**) and in the *Ly6g* + neutrophils (**D**). Analysis of the relative number of cells in each cluster expressing individual genes shows their distinct expression levels in violin plots for selected genes (**E**). Relative increases and decreases in the proportion of cells in the different clusters as a result of the progression of leukemia in the mouse cohorts are shown in (**F**), where the blue boxes highlight the increases seen in specific monocyte and neutrophil populations

While informative, the initial description of the major immune cell types in the PB using flow analysis, gave only a limited view of the overall changes in cellular composition in the microenvironment that might be occurring in response to the presence of the SCLL cells. These observations also do not provide any information about the gene expression reprogramming that accompanies the changes in cellular function. To investigate this immune modulation in more details, we performed scRNA-Seq analysis of leukocytes in the PB from BBC2 cell-engrafted mice after 11 (mid-stage leukemia) and 14 (late-stage leukemia) days compared with naive mice, where the flow cytometry results revealed changes in immune cell composition. PB samples were isolated from 3 different mice in each cohort and pooled for sequencing analysis to compensate for any inter-mouse variation. After exclusion of low-quality cells with high mitochondrial or globin gene expression and doublets, this analysis then resulted in ∼ 3000 cells per sample being included in a t-distributed stochastic neighbor enabling (t-SNE) algorithm analysis, which grouped the cells into 20 distinct clusters based on their shared gene expression profiles (Fig. 2A). Using the 5 most statistically significant genes from each cluster, a heatmap was generated as shown in Fig. 2B. In this analysis, distinct and often discrete subclassification was observed, for example in cluster 6. In other cases, there was overlap for some gene sets as seen in clusters 10 and 15, 18 and 19, for example, which might be expected since these represent closely related B-cell populations. The feature plots shown in Fig. 2C allow the visualization of relative gene expression levels of identified markers within the tSNE profile. In some cases, such as *Adgre4* and *Slc8a1*, expression is seen in different classes of monocytes, whereas *Pparg* seems more specific for the non-classical monocytes (ncMono). *Camp*, *Ngp*, *Ltf* and *Ly6g* are specific for the *Ly6g +* and *Camk1d +* neutrophils (Fig. 2D). A more quantitative assessment of the unique expression in terms of both number of cells expressing and the levels of expression can be seen in subsets of monocytes at different levels, although *Pparg* is specific to ncMono. Specific, high-level expression for *Camp* and *Ltf* is seen in the *Ly6g +* neutrophils. These observations, therefore, clearly define the different immune cell populations in the peripheral circulation and also identify associated genetic markers.

### Dynamic changes of immune cell composition during leukemia progression

As shown in Fig. 2A, the distribution of cells into the various clusters was consistent between the naive mouse control and the leukemic PB leukocytes. When the relative percentages of cells in each cluster was calculated, there were clearly some dramatic changes in the proportion of total cells present in some of these clusters, which are summarized in Fig. 2F. The presence of cells in cluster 4 is virtually absent in the naive mice (0.3%) but show a > 60-fold increase over 14 days in the BBC2 engrafted mice (18.2%). CIPR expression profiling identifies these cells as showing a mixed lineage gene profile including stem cell markers as well as T- and B-cell markers, which is consistent with the original description of BBC2 leukemia cells [5]. This connection is seen in the gene expression categorization for cluster 4 (Fig. 2B), which overlaps with the pre-B cell cluster 15 and is consistent with the arrested differentiation state of BBC2 cells [22]. Compared with normal B cells, high expression of cell proliferation markers such as *Mki67*, *Cdk6*, *Cdkn2a* and *Hist1h1b*, was noted in this cluster, indicating a BBC2 leukemia cell identity (Supplemental Fig. 2). Intriguingly, high mobility group box 1

(Hmgb1), a DNA chaperone protein that is overexpressed in tumor cells and can trigger inflammation, cell migration, and tumor metastasis, is also highly expressed in leukemia cells, suggesting a potential role in promoting leukemia progression [44].

Our flow cytometry data revealed an increase in both PMN-MDSC and M-MDSC during SCLL development, although the vast majority (> 50%) were PMN-MDSC. Unlike the flow cytometry definition of all PMN-MDSC mainly based on surface Ly6G protein expression [20, 45], scRNA-Seq divides the neutrophils into seven different clusters based on their gene expression profiles [45,46]. In fact, neutrophils showed the most diverse cell subtypes in the PB and are represented by clusters 0, 2, 3, 7, 9, 12 and 13, which were identified as different subclasses of neutrophils (Fig. 2A). We have annotated the different clusters based on the unique expression of individual specific genes within the neutrophil subgroups that were identified within the 10 most statistically significant markers in each group. Clusters 9 (*Camk1d +* Neutrophils) and 13 (*Ly6g +* neutrophils) show a close correlation with leukemia progression (Fig. 2F) with a progressive increase (> 10-fold) of *Ly6g* + neutrophils by Day 14 in BBC2 engrafted mice compared with naive mice (5.25% versus 0.47% respectively). These cells express high levels of *Ly6g* as well as genes such as *Ltf*, *Camp*, *Ngp*, *Ifitm6* and *Lcn2*, which have also been reported previously to be highly expressed in PMN-MDSC [45–47]. Most neutrophil clusters, however, including clusters 2 (*Retnlg* + neutrophils), 3 (*Cstdc4* + neutrophils) and 7 (*Rsad2* + neutrophils) maintained similar levels during leukemia progression, and two other neutrophil clusters, 0 (*Il1b* + neutrophils) and 12 (*Cxcr2* + neutrophils) showed a decrease in the percentage of cells (Fig. 2F).

The scRNA-Seq data also demonstrated that cells expressing the CD4 + T-cell marker (Fig. 2F) were observed in clusters 5 (*Cd4* + proliferative), 11 (*Cd4* + naive) and 17 (*Cd4* + active). All of these *Cd4* + T cell clusters showed dramatic reductions of relative percentages when compared between the naive and D14 leukemic mice. Similarly, Cd8 + expressing T-cells were identified in cluster 14, which also showed reductions in the comparison between naive and leukemic mice. In addition, cluster 16 showed cell reductions and were identified as Foxp3 + expressing T-regs, further confirming the role of MDSC, instead of Treg in immune suppression during leukemogenesis. Thus, there was a consistent reduction in all T-cell populations in the leukemic mice. Analysis of cluster 8, identified as NK cells, also showed a reduction in cell percentages in the leukemic mice. B-cells expressing the *Cd19* marker were identified in clusters 10 (B-mature), 18 (B-naive) and 19 (B-memory), which also show significant reductions in percentages in the leukemic mice compared with naive mice, although there was no change in the Pre-B-cells in cluster 15 (Fig. 2F). Thus, in tumor bearing mice, there is a significant overall reduction in all immune effector cells, which represents an immune suppressive TME promoting tumor cell growth and survival.

### Molecular characterization of the leukemia induced neutrophils

**Fig. 3:**
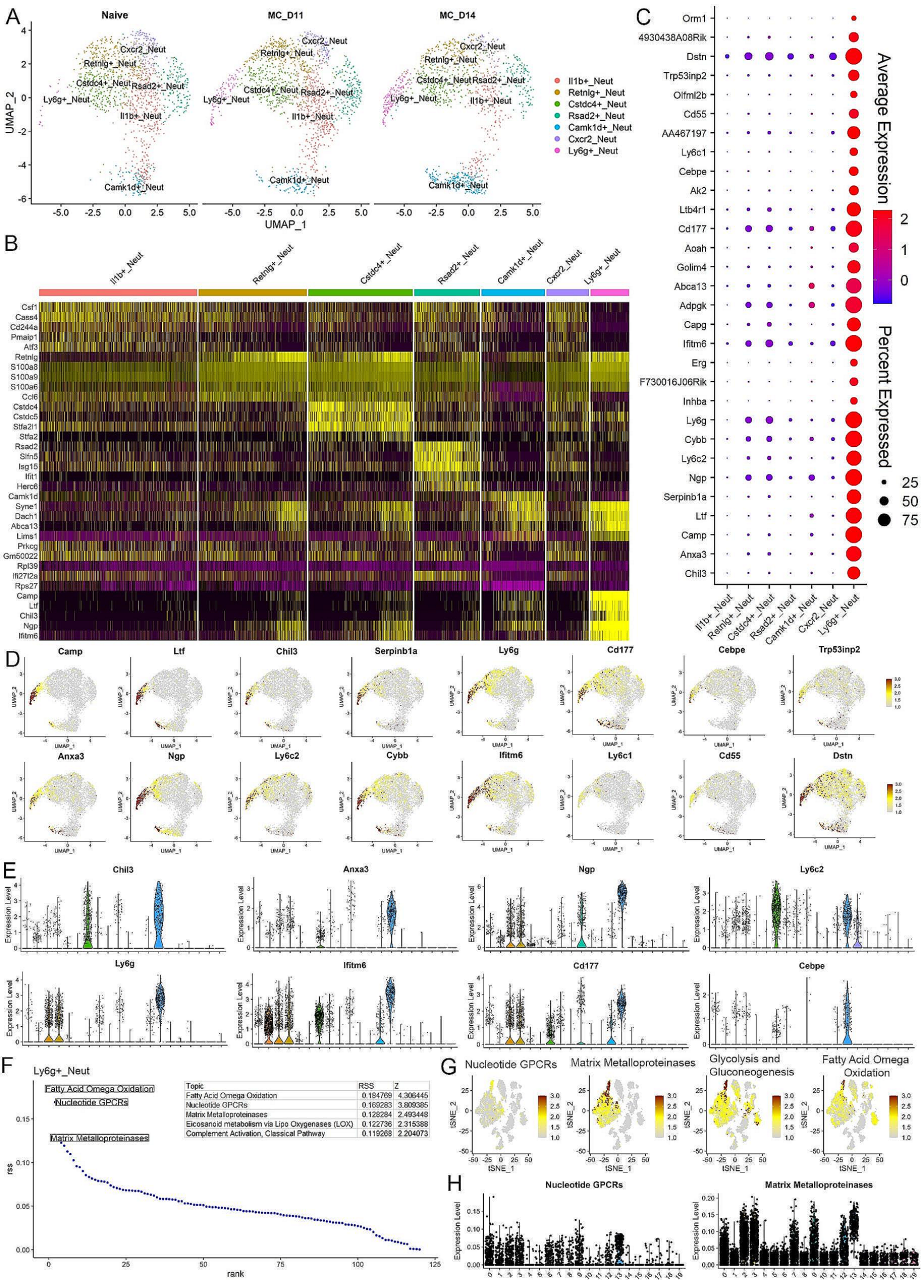
Molecular characterization of leukemia induced neutrophils. UMAP profile (**A**) of clusters specifically representing different subpopulations of neutrophils in mice engrafted with BBC2 cells (D11 and D14) compared with naive mice shows the dynamic changes of different subclusters, with predominant increase of the *Ly6g* + and *Camk1d* + subpopulations. The heatmap in (**B**) shows the top five markers identified in each subgroup based on the neutrophil-only analysis. While some profiles show the expression of identified markers is specific to the particular cluster, there is also overlap between closely related clusters. When the expression patterns of the 30 most statistically significant markers identified in the *Ly6g* + neutrophils are examined across all neutrophil cluster (**C**), most of these genes are highly specific for *Ly6g* + cells while some of them are expressed at lower levels in the other neutrophil clusters. The specificity of some of these identified markers for the *Ly6g* + cluster is shown in the feature plots in (**D**), with some displaying shared expression between the *Ly6g* + and *Camk1d* + clusters. Expression levels of selected markers across all clusters are shown in the violin plots in (**E**). Analysis of enriched Wikipathways in the *Ly6g* + cells identifies the five most upregulated pathways (**F**) including the matrix metalloproteinases. This preferential activation of the MMP pathway in the *Ly6g* + cells is confirmed in the feature plots (**G**) and violin plots (**H**)

Our flow cytometry data revealed PMN-MDSCs make up more than 50% of the total leukocytes in the peripheral circulation of leukemic mice (Fig. 1A). We have shown previously in our system that these cells are functionally active as immune suppressive cells [23]. To investigate the highly heterogeneous neutrophil population, we sub-grouped all the PMN-MDSC related neutrophils from the total data set for a focused analysis. Using the uniform manifold approximation and projection (UMAP) algorithm (Fig. 3A), the increases in the *Camk1d* + and *Ly6g* + cell clusters are clearly seen. Using the FindMarkers algorithm, we identified those genes uniquely expressed in each neutrophil subtype compared with all the other neutrophils (Supplemental Table 3), and the 5 most highly significant genes were plotted in the heatmap in Fig. 3B. It is interesting that the *Ly6g* + neutrophils show a relatively unique pattern, and although they share expression of some markers from *Retnlg* + and *Camk1d* + neutrophils, the markers for this cluster are mostly restricted to *Ly6g* + neutrophils (Fig. 3B).

It has been reported that the immune suppressive function of MDSCs is associated with specific gene signatures [47, 48]. The *Ly6g* + neutrophils show exclusive presence in leukemia mice and very likely represent a subgroup of the immunosuppressive neutrophils. The 30 most highly expressed genes identified are shown in the dot plot in Fig. 3C. While low-level expression of some of these genes was seen in other subgroups of neutrophils, in all cases the levels of expression and the consistency of expression in the population was far more exclusive in the *Ly6g* + neutrophils. For example, *Dstn*, *Cd177* and *Ifitm6* demonstrated somewhat shared expression across most of the neutrophil clusters, but genes such as *Erg*, *Inhba* and *Chil3* displayed a significant and often exclusive enrichment in high level gene expression in the *Ly6g* + cells (Fig. 3C). This unique, or predominant, expression in *Ly6g* + neutrophils is also highlighted in the feature plots shown in Fig. 3D and the violin plots in Fig. 3E.

Using the AUCell software package, we defined the highest probability pathways that were upregulated in all the clusters (Supplemental Tables 2 and Supplemental Fig. 3) and defined the 5 most highly enriched pathways in the *Ly6g* + neutrophils in leukemic mice as shown in Fig. 3F, where pathways involving the matrix metalloproteinases (MMPs) were highly prominent as well as pathways involving nucleotide GPCRs, glycolysis and gluconeogenesis and fatty acid omega oxidation, the latter three being related to cell metabolism. When these pathways were subjected to feature plot analysis across the 20 clusters, although low level expression was seen across all of the neutrophil subgroups (Fig. 3G), their high-level expression in the *Ly6g* + neutrophils was clear. The violin plots from this data further confirmed the increased activity of theMMP pathway in *Ly6g* + neutrophils (Fig. 3H).

### Leukemia induced transcriptional reprogramming in neutrophils

**Fig. 4:**
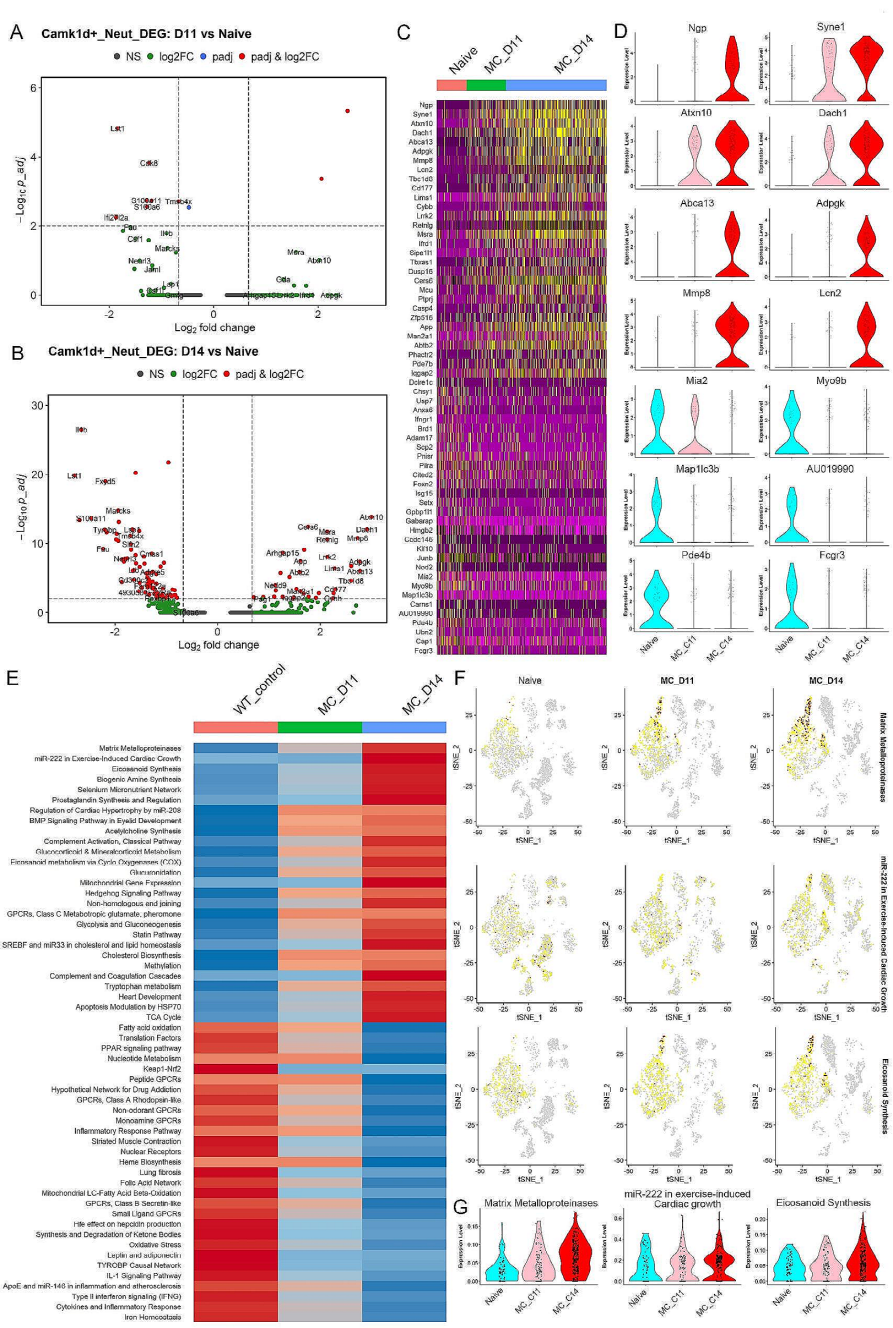
Transcriptional reprogramming in leukemia-induced neutrophils during leukemia development. Volcano plots show the differentially expressed genes (DEGs) in the *Camk1d* + neutrophils at Day 11 (**A**) and Day 14 (**B**), compared with their naïve counterparts. When these DEGs are plotted in a heatmap for expression levels (**C**), a clear view of upregulated and downregulated pattern is seen during leukemia progression. Examples of genes that appear to be specifically upregulated and downregulated as leukemia progresses is shown in the violin plots in (**D**). Analysis of the Wikipathways identified the matrix metalloproteinases pathway with the highest activation score during leukemia development (**E**). These observations are further confirmed in the feature (**F**) and violin (**G**) plots.

To gain a better understanding of the biological function of the leukemia induced neutrophils, we analysed the differential gene expression during leukemia progression, focusing on the *Ly6g +* and *Camk1d +* neutrophils that showed increased levels in the leukemic mice. Unfortunately, the rare presence of *Ly6g +* neutrophils in the naïve mice precluded obtaining sufficient statistical power to perform this analysis but potential candidate genes were defined (Supplemental Fig. 4). Considering the continuous changes in gene expression during leukemia progression, we also compared gene expression in Ly6g + neutrophils between D11 and D14 where a higher number of genes with significant differential expression were identified (Supplemental Fig. 4C). Moreover, the *Camk1d* + neutrophils showed an extensive and progressive reprogramming of gene expression in cells from the D11 and D14 leukemic mice as shown in the volcano plots (Fig. 4A and B). This reprogramming is highlighted in the heatmap depicting the 30 most highly activated and inactivated genes in a comparison between D11/D14 leukemic mice and naïve mice (Fig. 4C). Of the upregulated genes (Fig. 4D), *Syne1*, *Atxn10* and *Dach1* show activation as early as Day 11, while *Ngp*, *Abca13*, *Adpgk*, *Mmp8* and *Lcn2* were more significantly upregulated in the later stage, D14 neutrophils,which suggests a sequential activation in *Camk1d +* neutrophils during leukemia progression. Similarly, *Fcgr3*, *Pde4b*, *Map1lc3b*, *Au109990*, *Myo9b* and *Mia2* are inactivated (Fig. 4D) in the *Camk1d +* neutrophils during leukemogenesis, with only *Mia2* showing a less rapid inactivation.

The relative activities of different pathways in the *Camk1d* + neutrophils were processed through the AUCell Pathways algorithm, and pathways showing continuous increase or decrease during leukemogenesis were identified. The heatmap in Fig. 4E displays the 30 most activated or inactivated pathways in the *Camk1d* + neutrophils, with MMPs being the most highly activated pathway during leukemia progression. This pathway was also highly enriched in the *Ly6g* + neutrophils from leukemic mice (Fig. 3F and Supplemental Fig. 3), implying a potential role in immune suppressive PMN-MDSC. In addition, this MMP pathway shows remarkable specific activation during leukemia progression, compared with the miR-222 in exercise induced cardiac growth and eicosanoid synthesis pathways (Fig. 4F and G). In summary, these leukemia induced *Camk1d* + neutrophils undergo transcriptional reprogramming, and the differentially expressed genes and pathways identified may be associated with their function in immune suppression.

### ***Ly6g*+ Neutrophils are the potential neutrophil progenitors**

**Fig. 5:**
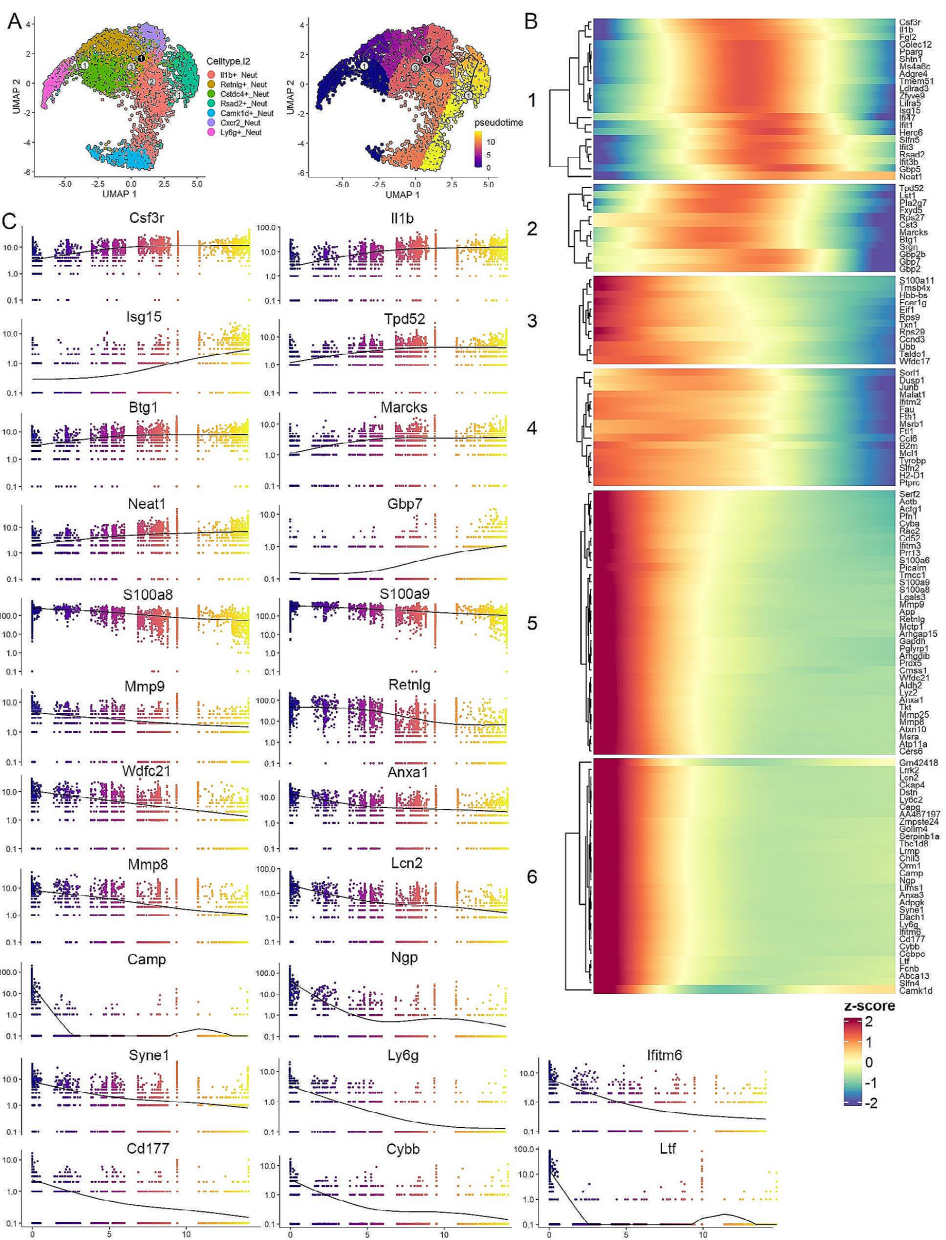
Trajectory analysis of the potential differentiation path of leukemia induced neutrophils. UMAP distinction of neutrophil populations is shown in (**A**, left). Pseudotime estimates of differentiation waves across the neutrophil populations is shown in (**A**, right), where the progress is tracked from the *Ly6g* + neutrophils across various subpopulations. The branchpoint is shown in the black filled ‘1’ and branches are then defined as 1–3 marked with gray circles. The identified module genes across the pseudotime continuum are subdivided into 6 subgroups depending on the patterns of gene expression changes (**B**). An analysis of the variance of specific genes in individual cells using RSS are presented in (**C**), with the color coding defining the timing of expression changes across the pseudotime continuum.

To gain a better understanding of the development of neutrophils in the PB during leukemogenesis, we performed a trajectory analysis that sought to order cells along a lineage using a pseudotime estimate based on their gene expression profiles. As shown in Fig. 5A, the UMAP analysis defines the relative relationship between the different neutrophil subgroups, where the *Ly6g* + neutrophils are assigned as the root and least differentiated cells. As differentiation proceeds, the transition point during the differentiation continuum is defined within the *Retnlg* + neutrophils, and the branch points from here lead into the *Rsad2*+, *Cstdc4* + and *Il1b* + neutrophils (Fig. 5A). The modulated genes that are associated with neutrophil differentiation were identified (Supplemental Table 4), and a heatmap was generated using the K means method to show the gene expression changes across the continuum, which defined 6 subgroups depending on the expression pattern during the differentiation process (Fig. 5B). This data defines genes that are upregulated as the *Ly6g* + cells differentiate as well as genes that are downregulated during this process, where those downregulated constituted the majority of genes. As shown in groups 5 and 6, specific genes are downregulated in contrast to that seen in groups 3 and 4, where there is a progressive reduction in expression levels, but which still retain low level expression. The genes in groups 1 and 2, however, are transiently activated at different stages in the continuum, although becoming reduced to lower-level activity in the final stages. The data presented in Fig. 5B are based on the z score across all cells in the different subgroups. When these are plotted using residual sum of the squares (RSS) analysis, the between-cell variance in the dataset is taken into consideration, which presents a clearer picture of the up- (e.g. *Il1b*, *Isg15*, *Csf3r* and *Gpb7*) and down-regulation (e.g. *Cybb*, *Ly6g*, *Ngp*, *Ifitm6* and *Ltf*) of specific genes (Fig. 5C) during the predicted differentiation process.

### Matrix metalloproteinases activation in neutrophils and targeted disruption

**Fig. 6:**
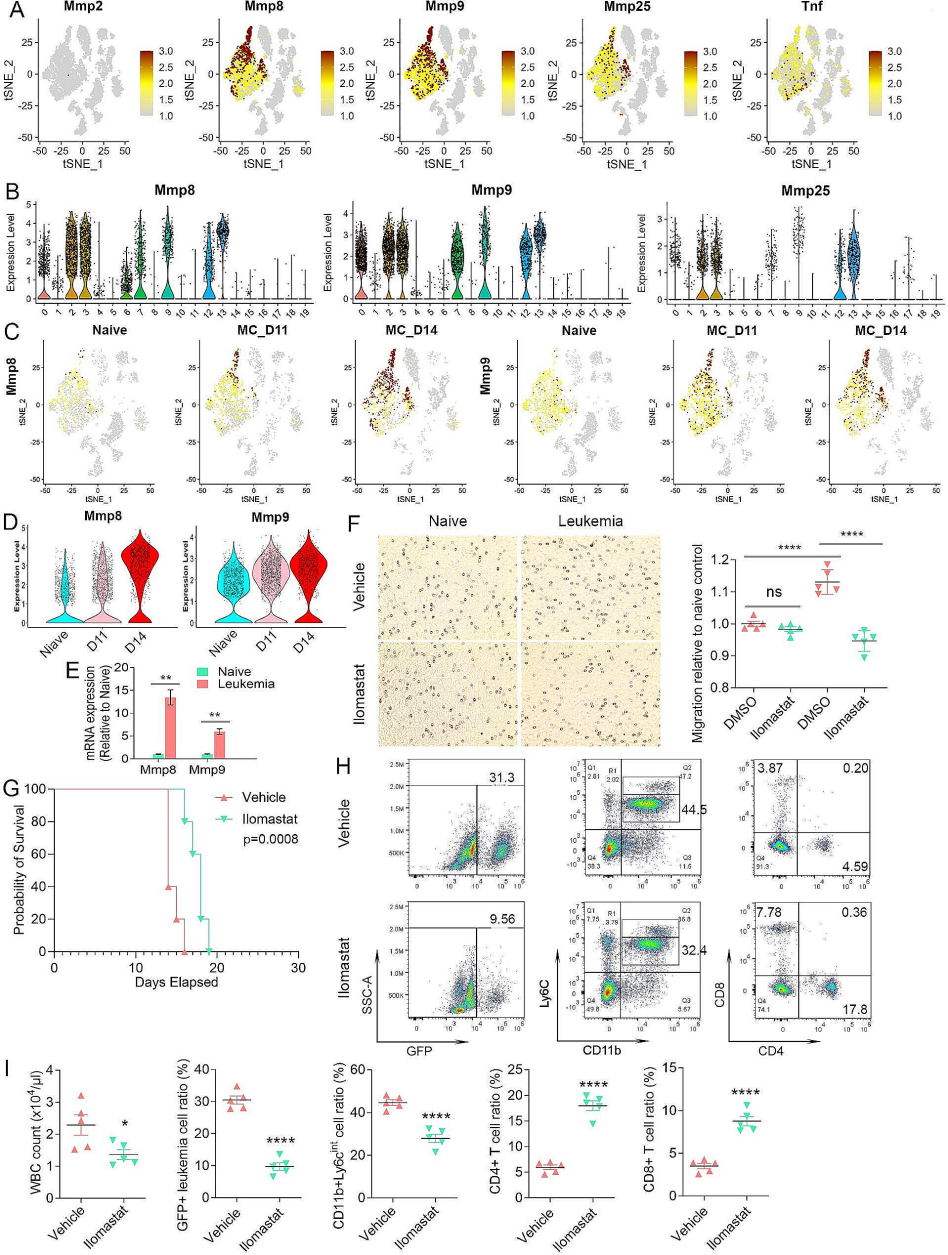
MMP pathway genes are involved in neutrophil mobilization and recruitment. Feature plots of gene expression of the *Mmps* in the TME of leukemic mice (**A**) show absence of expression of *Mmp2* throughout and increased expression of *Mmp8*, *9* and *25* in different neutrophil populations, with predominant expression in the *Ly6g* + and *Camk1d* + neutrophils. In contrast, high-level *Tnf* expression is mostly restricted to the *Il1b* + neutrophils. The relative expression levels of *Mmp8*, *9* and *25* across the TME clusters is shown in the violin plots in (**B**). Feature plots for *Mmp8* and *9* during leukemogenesis are shown in (**C**) and expression levels for *Mmp8* and *9* during leukemia progression are shown in the violin plots in (**D**). Expression levels of *Mmp8* and *Mmp9* (*N* = 3) as determined by qPCR (**E**) shows significantly increased expression levels in neutrophils from the leukemic mice compared with naive mice. From in vitro migration assays, Cystal Violet staining (F, left) shows that neutrophils from leukemic mice show significantly higher cell motility compared with naïve neutrophils. In addition, while Ilomastat treatment does not affect isolated neutrophils from naive mice, neutrophils from leukemic mice show significantly decreased migration compared with DMSO-treated cells. The migration levels for each cohort (*n* = 5) is shown in (**F**, right). Kaplan-Meier analysis of survival in vivo shows that leukemic mice treated with Ilomastat have a significantly increased survival compared with vehicle control treated mice (**G**). Flow cytometric analysis (**H**) shows a reduction in GFP + leukemic cells in Ilomastat treated mice with a concomitant increase in CD4+/CD8 + T-cells and a decrease in CD11b + Ly6C^Int^ PMN-MDSCs. Levels of these cells from all five mice in each cohort are shown in (**I**)

The most consistently upregulated pathway seen in the two leukemia responsive neutrophil populations that show increased percentages in the presence of leukemia involved the MMPs (Figs. 3F and 4E), implying potential involvement in MDSC mediated immune suppression. A feature plot of all *Mmps*(Supplemental Fig. 5) demonstrates that only *Mmp8*, *9*, and *25* are highly expressed in different subgroups of neutrophils, although mainly in the *Ly6g* + neutrophils (Fig. 6A). *Tnf*, which is also part of the MMP pathway, is much less active. The relative expression levels across the 20 different clusters from the PB are also shown in violin plots (Fig. 6B). The feature plots in Fig. 6C show the increasing expression of *Mmp8* and *Mmp9* in the *Ly6g* + neutrophils during leukemogenesis, which was further confirmed by violin plot of *Ly6g* + neutrophils at different stages (Fig. 6D). RT-PCR analysis of PB neutrophils from naive mice and D14 leukemic mice confirmed significant increased expression for *Mmp8* and *Mmp9* (Fig. 6E). Interestingly, the high-level expression of *Mmp8* and *Mmp9*, originally seen in *Ly6g +* neutrophils, has now extended to other neutrophil subpopulations by D14 (Fig. 6C), supporting the trajectory prediction that *Ly6g* + cells are neutrophil progenitors. A progressive downregulation of these *Mmps*, however, can be seen in the pseudotime continuum as the differentiation process proceeds (Fig. 5B).

MMP8 and MMP9 [49–52] release has been shown to promote stem cell mobilization in the BM and are derived from granulocytic neutrophils. It is possible, therefore, that the increased expression in the immature *Ly6g* + neutrophils appears to result from this increased mobilization and recruitment of PMN-MDSC progenitors to the peripheral circulation in the presence of leukemia. To address this hypothesis, we isolated Ly6G + neutrophils from the PB neutrophils from naive and D14 leukemic mice, and seeded them in migration chambers in the presence of either DMSO or the broad spectrum MMP inhibitor, Ilomastat [38, 39]. In the DMSO group, migration of neutrophils was significantly higher in the cells isolated from the leukemic mice (Fig. 6F) compared with those from naive mice. While the inhibitor did not affect migration potential of neutrophils from naive mice, there was a significant reduction in cell migration ability of neutrophils derived from leukemic mice (Fig. 6F). These in vitro observations were then extended to in vivo studies (Fig. 6G), where leukemic mice treated with the MMP inhibitor led to a significant improvement in survival, which correlated with a reduced WBC count. Flow cytometric analysis demonstrated that leukemic mice treated with inhibitor showed a significant reduction in GFP + leukemic cells in the PB (Fig. 6H and I), which correlated with increased levels of CD4 + and CD8 + T-cells and a reduction in the levels of PMN-MDSC or leukemia induced neutrophils. These observations support the idea that high MMP expression in leukemia-induced neutrophils from the BM promotes their mobilization to the PB in the presence of leukemic cells, which accounts for the accumulation of PMN-MDSCs or TANs and subsequent suppression of antitumor immunity.

### MMP8 can serve as an independent indicator of neutrophil infiltration and cancer prognosis

**Fig. 7:**
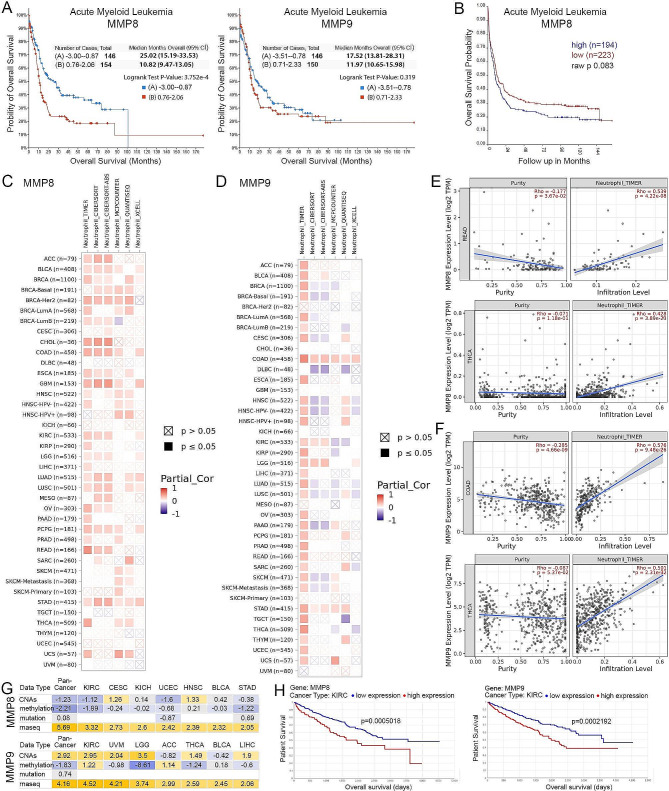
Clinical relevance of *MMP8* and *MMP9* expression in leukemia and solid tumors. Kaplan-Meier analysis of the OHSU AML cohort in TCGA shows a highly significant increase in survival for patients with lower expression levels of *MMP8* (**A**, left) but not for *MMP9* (**A**, right). The observations for *MMP8* are confirmed in an independent AML data set (**B**). In an analysis of data from 40 solid tumors in the TCGA dataset using the TIMER2.0 algorithm, 26 show a significant positive correlation between *MMP8* expression and tumor neutrophil infiltration levels. These observations are largely confirmed using five other algorithms (**C**). *MMP9* expression levels show a similar association with neutrophil infiltration using the TIMER algorithm but show less consistency when other algorithms are used (**D**). Examples of significant correlations between *MMP8* expression levels and neutrophil infiltration levels using READ and THCA datasets (**E**) as well as for *MMP9* using COAD and THCA datasets (**F**). When the purity levels of neutrophils in these samples is examined, there are either no, or less significant, correlations between *MMP8* and *MMP9* expression (**E**, **F**) while the infiltration levels show highly significant correlations. In an analysis of different types of genetic changes involving *MMP8* or *MMP9* in different tumor types, only expression levels determined by RNA-Seq show a significant correlation with poor prognosis (**G**). Negative correlations are shown in blue and positive correlations in yellow. In this analysis the Z scores shown are calculated from Cox univariate hazards models, regressing a gene or genetic feature against patient outcome in the indicated cancer type. A Z-score > 2 is equivalent to a *p*-value < 0.05. This is confirmed in Kaplan-Meier analysis where high *MMP8* and *MMP9* expression levels are associated with poor survival in KIRC as an example (**H**). ACC, Adrenocortical carcinoma; BLCA, Bladder Urothelial Carcinoma; BRCA, Breast invasive carcinoma; CESC, Cervical squamous cell carcinoma and endocervical adenocarcinoma; CHOL, Cholangiocarcinoma; COAD, Colon adenocarcinoma; DLBC, Lymphoid Neoplasm Diffuse Large B-cell Lymphoma; ESCA, Esophageal carcinoma; GBM, Glioblastoma multiforme; HNSC, Head and Neck squamous cell carcinoma; KICH, Kidney Chromophobe; KIRC, Kidney renal clear cell carcinoma; KIRP, Kidney renal papillary cell carcinoma; LGG, Brain Lower Grade Glioma; LIHC, Liver hepatocellular carcinoma; LUAD, Lung adenocarcinoma; LUSC, Lung squamous cell carcinoma; MESO, Mesothelioma; OV, Ovarian serous cystadenocarcinoma; PAAD, Pancreatic adenocarcinoma; PCPG, Pheochromocytoma and Paraganglioma; PRAD, Prostate adenocarcinoma; READ, Rectum adenocarcinoma; SARC, Sarcoma; SKCM, Skin Cutaneous Melanoma; STAD, Stomach adenocarcinoma; TGCT, Testicular Germ Cell Tumors; THCA, Thyroid carcinoma; THYM, Thymoma; UCEC, Uterine Corpus Endometrial Carcinoma; UCS, Uterine Carcinosarcoma; UVM, Uveal Melanoma

To examine the clinical relevance of the MMPs identified here, we first determined whether *MMP8* and *MMP9* expression is associated with clinical outcome in leukemia patients. Using the extensive OHSU AML dataset in TCGA [30], we demonstrated that high *MMP8* expression, but not *MMP9* expression, was associated with significantly poor survival (Fig. 7A), which was confirmed (Fig. 7B) in an independent AML dataset using the R2 platform [40]. In the OHSU dataset, patients with low *MMP8* expression have a median survival of 25.02 months, compared to 10.82 months in the *MMP8* highly expressing group. The clinical significance of *MMP8* was further confirmed by a multivariate survival analysis using the Cox proportional hazard model, with the hazard ratio (HR) 1.465 for *MMP8* in the high expression group compared to the low expression group with a 95% confidence interval (CI) [1.070, 2.005], which is statistically significant with *p* = 0.017 (Supplemental Table 5). Considering the mixed presence of SCLL, we also performed a correlative analysis in a publicly available data set for pediatric ALL patients [29]. Even though the results did not reach the significance threshold of 0.05 due to limitation in sample size, there is still a clear separation of survival between the patients showing high and low levels of *MMP8* and *9* expression (Supplemental Fig. 6A). The mean survival further confirmed the benefit of low expression levels of *MMP8* and *9*, with the unreached survival end point for *MMP8* lowly expressed group and a doubled survival for the *MMP9* lowly expressed group. These data demonstrate that genes discovered in our SCLL model show clinical relevance in a broader study of both AML and pediatric ALL patients and confirmed the efficiency of *MMP8* as an indicator for clinical prognosis of leukemia patients.

Considering the possibility that the genes identified in leukemia induced neutrophils can play similar roles in TANs from other cancer types, we performed Tumor Immune Estimation Resource (TIMER)2.0 analysis [41] to predict associations between *MMP8* or *MMP9* expression and neutrophil infiltration using available datasets in The Cancer Genome Atlas (TCGA). Even though the leukemia data is not available in this platform, the TIMER algorithm detected a positive correlation between *MMP8* expression and neutrophil infiltration in 26 of the 40 cancer types examined, most of which were validated using multiple different algorithms (Fig. 7C). In contrast, *MMP9* also shows an association with neutrophil infiltration but was less consistent among the different algorithms (Fig. 7D). The correlation plots from two cancer types with the highest Rho values (Spearman’s rank-order correlation coefficient) are shown in Fig. 7E. While there is either no significant or a less significant association between gene expression and neutrophil purity, there is consistently a highly significant association between *MMP8* or *9* expression and the level of neutrophil infiltration, indicating a conserved role for *MMP8* or *9* in neutrophil mobilization and recruitment in multiple cancers. Since *MMP8* and *9* were identified in our SCLL models, driven by over activated FGFR1 signaling, we further examined the association between *FGFR1* expression levels and neutrophil infiltration in KIRC and found a strong positive correlation. This correlation is not only present for the *FGFR1* gene, but also for *IRAK1*, which is an important downstream target of FGFR1 fusion kinases involved in immune modulation (Supplemental Fig. 6B) [23].

The correlation between *MMP8* or *9* expression levels and survival was examined in the TCGA databases using the TCGA-survival platform [42], which provides access to a comprehensive analysis of gene mutations, copy number alterations, methylation, microRNA, mRNA, and protein expression patterns linked with cancer patient outcome. As shown in Fig. 7G, the Z scores were calculated from Cox univariate hazard models regressing a gene or genetic feature against patient outcome in the indicated cancer type. Z scores of > 2 indicates a *P* value < 0.05. It is clear that *MMP8* or *9* mRNA expression is most significantly associated with cancer patient survival and the most significant correlation is observed in the Kidney Renal Clear Cell Carcinoma (KIRC), as shown in Fig. 7H. The clinical significance of *MMP8* was further confirmed by a multivariate survival analysis in KIRC patient samples (Supplemental Table 5). A similar significant association has been identified for *IRAK1*, which is reported by our group with a critical function in the recruitment of PMN-MDSC or neutrophils (Supplemental Fig. 6C). These results clearly demonstrate that even though there are some potential differences between leukemias and solid tumors, the genes identified in leukemia induced neutrophils may also be of compelling clinical value and significance for solid tumors.

## Discussion

With the advent of single cell sequencing, it is now possible to define individual subsets of cell types in the TME based on their differential gene expression profiles [53–56]. This approach is being used to define the biology and genetics of MDSC and how they promote tumorigenesis through suppressing the immune system [45, 57]. These studies are also defining specific genetic targets that might be used to suppress MDSC development and overcome immune tolerance [45, 57, 58]. To date, the vast majority of these kinds of studies, however, have centered on the MDSC within solid tumors, which have infiltrated into the tumor mass. Leukemias, however, have a very different pathobiology, since they coexist in the circulatory system with the immune cells. Lymphomas on the other hand, are more akin to solid tumors because of their localized tumor masses [59]. It might be expected, therefore, that immune cell responses to leukemias differ in terms of the genetic reprogramming that is occurring in solid tumors [59]. While there have been some studies of MDSC in leukemias in both mouse [23, 60] and human [59], these have typically involved flow cytometric analyses characterizing cells based on broad expression of predetermined cell surface markers. These studies have demonstrated increased levels of MDSC and reductions in immune effector cells in the leukemia samples. The studies reported here, however, possibly for the first time, have characterized all cell types in the leukemic circulatory TME during leukemogenesis and provided insights into real time transcriptional changes that are occurring within them. In particular, we have defined specific subclasses of TANs which we have defined by the unique expression of representative genes within each subgroup. This analysis has demonstrated that, in response to leukemogenesis, two specific subgroups of *Ly6g* + and *Camk1d* + neutrophils show significantly increased levels in leukemic mice and are presumed to be the PMN-MDSC precursor cells. Pseudotime analysis also predicts how these precursors progress and mature within the TME as the tumor evolves. The co-expression of *Npg*, *Cd177* and *Cybb* in the SCLL *Ly6g* + and *Camk1d* + neutrophils suggested that they are closely related, which is consistent with both being increased in tumor bearing mice. Of particular significance is that, unlike previous studies that have presorted Ly6G + PMN-MDSC [45], we have performed an unbiased analysis of all cell types, which has allowed us to define distinct subsets of neutrophils as well as their potential effects on other immune cells in the TME.

The FGFR1 transformation of hematopoietic stem cell models that we have developed have provided a unique opportunity to study the total cellular changes occurring in the microenvironment during leukemogenesis, which is not afforded by the analysis of human leukemias. Importantly, the widely used PB and BM mononuclear cell preparation methods currently in clinical practice are based on density gradient separation, which will remove granulocytes (including PMN-MDSCs/neutrophils). Current cryopreservation procedures are also not efficient in preserving the highly fragile PMN-MDSCs/neutrophils [61]. Our mouse model, however, does not suffer from these shortcomings. The scRNA-Seq with fresh PB samples provides a global profiling of PMN-MDSCs at different stages of leukemogenesis, allowing an investigation of the transcriptional reprogramming in these cells accompanied by the establishment of immune suppression.

The *Ly6g* + and *Camk1d* + neutrophils in our study show a distinct pattern of gene expression within the overall neutrophil populations, possibly defining genes that are important in the early development of PMN-MDSC before they mature. While ∼ 50% of the genes (e.g., *Npg*, *Camp, Ltf*, *Chil3*, *Cd177*) showing high-level expression in these early-stage leukemia related MDSC, have also been reported in MDSC in other setting, such as lung cancer [45], the remaining 50% appear to be unique to the SCLL TME (e.g., *Erg*, *Capg*, *Golim4*, *Cd55*, *Trp53inp2*). These observations may be important if gene expression in MDSC is to be considered a means of stratifying cancer patients or even in choosing molecular targets for therapy. If indeed different tumors induce a different TME, this might become particularly problematic in AML since there are more than 50 cytogenetic subgroups [62], where subtle differences in gene expression may be important in designing markers and defining targets. Therefore, successful identification of conserved molecular events holds the key to developing efficient immunotherapy with broad applications.

A consistent finding in the two leukemia-responsive neutrophil subgroups, which show increased levels in response to the presence of leukemic cells, was high-level expression of specific *MMPs*. Of the 24 *MMP* genes in the mammalian genome, only *Mmp8*, *9* and *25* were expressed in the *Ly6g* + and *Camk1d* + neutrophils, all of which were down regulated during maturation as shown in the pseudotime analysis. The MMP8 collagenase and MMP9 gelatinase are secreted by neutrophil precursors in the BM, allowing their release from the BM niche into the peripheral circulation [49–52]. MMP25 differs from MMP8 and 9 in being a membrane bound MMPs, which was shown to be expressed exclusively in PB leukocytes [63] and PMNs in particular [64, 65]. MMP25, however, can be mobilized and secreted into the extracellular milieu and can target basement membrane proteins [66], which has also been implicated in cell migration and invasion. MMP8 and MMP9 release has been shown to promote stem cell mobilization in the BM and are derived from granulocytic neutrophils [67]. It is possible, therefore, that the increase in the *Ly6g* + and *Camk1d* + immature neutrophils is a result of this increased mobilization in the presence of leukemia. When treated with an MMP inhibitor, the number of Ly6G + neutrophils or PMN-MDSCs in the PB is reduced in vivo and a reduced ability of these neutrophils to migrate in vitro supports this idea. Consistent with this hypothesis, several other genes, such as *Pfn1*, *Cd52*, *Anxa1*, *Fau*, *Marcks* and *Cd177*, which are related to cell movement and migration, are also highly expressed in the *Ly6g* + neutrophils and subsequently down regulated on maturation. Reduction of these neutrophil populations in the PB following pharmacological suppression of MMP function supports their role as PMN-MDSCs in the PB, which accompanies increased levels of T-cells, prolonged survival and attenuated leukemogenesis.

The demonstration of the role of specific MMPs in our preclinical leukemia model and the subsequent correlation with AML patient outcome supports the discovery value of these mouse leukemia models. While most published work on MMPs has been focused on the role of MMP9 in neutrophil release, our study revealed an underappreciated role for MMP8 in TANs. Mining clinical data from solid tumors demonstrated that MMP8 can not only predict neutrophil infiltration in most cancer types but were also significantly associated with cancer patient outcomes, demonstrating that MMP8 in particular is a conserved features of cancer induced neutrophils with high clinical significance. It is perhaps not surprising that MMP9 failed to match the performance of MMP8 in predicting neutrophil infiltration, since MMP9 is widely expressed at low levels in neutrophils from naïve mice, while MMP8 is specifically activated in leukemic mice. These observations, however, suggest that the MMP pathway may be an attractive target for blocking PMN-MDSC recruitment in attempts to restore antitumor immunity not only in leukemia, but also in a broad spectrum of other cancers.

## Conclusions

Mouse models of cancer where they recapitulate the phenotypes and genotypes of human disease provide a unique opportunity to study changes that occur in the immune system as a result of the development of cancer. Here we have dissected the diverse cellular changes that occur in the TME during the development of leukemia and in particular define distinct subgroups of neutrophils and explore their relationship with the development of PMN-MDSC. Through defining the transcriptional reprogramming within these cells, we provide a foundational report of genes associated with leukemia related events in the TME which suggests a mechanism whereby *Ly6g* + precursor neutrophils are released from the bone marrow through upregulation of MMPs. The mouse model has also provided for a preclinical evaluation of the possibility of targeting this release of neutrophil precursor cells to reduce their ability to promote immune tolerance.

### Electronic supplementary material

Below is the link to the electronic supplementary material.

**Supplementary Material 1: Supplemental Fig. 1.** **Flow cytometry monitoring of the major immune cells in the BCRF8C and ZNF112 mouse models.**
Representative flow diagrams of PB samples from mice engrafted with BCRF8C (**A**) and ZNF112 (**B**) SCLL cells during leukemogenesis. Levels of CD4 + and CD8 + T-cells, Ly6C + CD11b + myeloid cells as well as CD19 + B-cells and CD49b + NK cells are shown from the PB, over 21 days (D7-D21) for BCRF8C and over 27 days (D14-D27) for ZNF112 cells. A progressive increase in Ly6C + CD11b + cells is seen in both models and all immune effector cells show a decrease. While the Ly6C^hi^CD11b + cell population (M-MDSC) shows only a modest increase, the proportion of Ly6C^int^D11b + PMN-MDSC show a highly significant increase. Leukemic cells are defined by the expression of GFP. The cell counts of each individual cell type included in this analysis are shown in (**C**) for BBC2 and (**D**) for ZNF112.

**Supplementary Material 2: Supplemental Fig. 2.** **Feature plots for gene defining B cell progenitor and cell proliferation in leukemia cells.**
Feature plots of genes highly expressed in the leukemia cell cluster show genes that are also expressed in pre-B cells (e.g. *Vpreb3*, *Vpreb1*, *Ebf*). In addition, genes relating to active cell cycling in proliferating leukemic cells including, *Cdkn2a*, *Cdk6*, *Hist1a1b*, and *Mki67*, are shown in the feature plots.

**Supplementary Material 3: Supplemental Fig. 3.** **Most significant wikipathways activated in different cell subtypes.**
Summary of most active pathways across the 20 clusters depicted in a heatmap in (**A**). Violin plots for selected pathways (**B**).

**Supplementary Material 4: Supplemental Fig. 4.Identification of the differentially expressed genes in the leukemia induced Ly6g+ neutrophils.** Volcano plots show differentially expressed genes (DEGs) in *Ly6g* + neutrophils at D11 (**A**) and D14 (**B**) compared with naïve mice. A comparison between D11 and D14 leukemic mice is shown in (**C**). A heatmap (**D**) of gene expression level changes over the D0 (naive)-D14 period during leukemogenesis shows the individual genes upregulated (from blue to red) compared with those downregulated (from red to blue). Violin plots for the 8 most upregulated and 6 most downregulated genes are shown in (**E**).

**Supplementary Material 5: Supplemental Fig. 5.** **Expression levels of MMP genes in different cell subtypes.**
Feature plots for *Mmp* genes shows only *Mmp8*, *9* and *25* are expressed at high levels in cells from the PB TME of D14 leukemic mice. Expression levels of *Timp2* and *Tnf*, which are members of the MMP Wikipathway, are also shown (**A**). The violin plots shown in (**B**) demonstrate the expression levels of all *Mmp* genes in the 20 clusters identified in the PB TME.

**Supplementary Material 6: Supplemental Fig. 6.** **Clinical relevance of target genes in ALL and KIRC.**
Kaplan-Meier analysis of the Pediatric Acute Lymphoid Leukemia (ALL) in Phase II clinical trial cohort shows decreased survival in patients with high-level expression of *MMP8* (**A**, left) and *MMP9* (**A**, right). In an analysis of KIRC patient data in the TCGA dataset using the TIMER2.0 algorithm, there is a significant positive correlation between *FGFR1*expression and tumor neutrophil infiltration levels (**B**, left). Similarly, a significant positive correlation between *IRAK1*expression and tumor neutrophil infiltration levels is also detected in this data set (**B**, right). Kaplan-Meier analysis for IRAK1 expression levels in KIRC patients (**C**) shows poorer survival in patients expressing high levels of IRAK1.

**Supplementary Material 7: Supplemental table 1**
Markers identified in different clusters using the FindMarker function. avg_logFC: log fold-change of the average expression between cells in the target cluster and cells from the remaining clusters. Positive values indicate that the gene is more highly expressed in the target cluster. pct.1: The percentage of cells where the gene is detected in the target cluster. pct.2: The percentage of cells where the gene is detected in the remaining clusters. p_val_adj: Adjusted *p*-value, based on Bonferroni correction using all genes in the dataset.

**Supplementary Material 8: Supplemental table 2**
The most significant Wikipathways in different clusters identified using the AUCell package in the SCENIC workflow. The relative activities of indicated pathways are calculated based on the proportion of expressed genes in the signature and their relative expression value compared to the other genes within the cell.

**Supplementary Material 9: Supplemental table 3**
Markers identified in different neutrophil subclusters using FindMarker function. avg_logFC: log fold-change of the average expression between neutrophils in the target cluster and neutrophils from all the remaining clusters. Positive values indicate that the gene is more highly expressed in the target cluster. pct.1: The percentage of neutrophils where the gene is detected in the target cluster. pct.2: The percentage of neutrophils where the gene is detected in the remaining clusters. p_val_adj: Adjusted *p*-value, based on Bonferroni correction using all genes in the dataset.

**Supplementary Material 10: Supplemental table 4**
Modulated genes identified using the Monocle 3 workflow, whose expression is changing in a continuous manner over pseudotime.

**Supplementary Material 11: Supplemental Table 5.**
Multivariate analysis of *MMP8* and *9* in AML and KIRC using Cox proportional hazard ratio model for overall survival. From the multivariate survival analysis accounting for age, sex, and disease stage, the hazard ratio (HR) for *MMP8* in AML is 1.465 in the high expression group compared to the low expression group with 95% CI [1.070, 2.005], which is statistically significant with *p* = 0.017; the HR for *MMP8* in renal cell carcinoma is 1.556 in the high expression group compared to the low expression group with 95% confidence interval (CI) [1.136, 2.131], which is statistically significant with *p* = 0.00589; the HR for *MMP9* in AML is 1.025 in the high expression group compared to the low expression group with 95% CI [0.756, 1.390], which is not statistically significant with *p* = 0.873; the HR for *MMP9* in renal cell carcinoma is 1.365 in the high expression group compared to the low expression group with 95% CI [0.996, 1.870] with borderline significance *p* = 0.053.

## Data Availability

All data generated or analysed during this study are included in this published article and its supplementary information files.

## Abbreviations

AML: acute myelogenous leukemia
AUC: area under the curve
BEAM: Branched Expression Analysis Modeling
BM: bone marrow
CIPR: Cluster Identity Predictor
DFpANN: DoubletFinder Artificial nearest neighbor
FC: fold change
FGFR1: fibroblast growth factor receptor 1
GPCRs: G protein-coupled receptors
GFP: green fluorescent protein
MDSC: myeloid-derived suppressor cells
M: MDSC-monocytic MDSC
MIR: microRNA
MMP: matrix metalloproteinase
NK: natural killer
OHSU: Ohio State University
PB: peripheral blood
PMN: MDSC-polymorphonuclear MDSC
PD: L1-programed cell death like 1
RSS: residual sum of the squares
scRNA Seq: single-cell RNA sequencing
SCLL: Stem Cell Leukemia/Lymphoma syndrome
tSNE: t-distributed stochastic neighbor embedding
TAN: tumor-associated neutrophils
TCGA: The Cancer Genome Atlas
TME: tumor microenvironment
TIMER: Tumor immune estimation resource
UMAP: uniform manifold approximation and projection
