# Supplementary figures and images for: Single-cell analysis defines highly specific leukemia-induced neutrophils and links MMP8 expression to recruitment of tumor associated neutrophils during FGFR1 driven leukemogenesis
Source: Exp Hematol Oncol. 2024 May 10;13:49. doi: 10.1186/s40164-024-00514-6 (PMC11084112; doi:10.1186/s40164-024-00514-6)

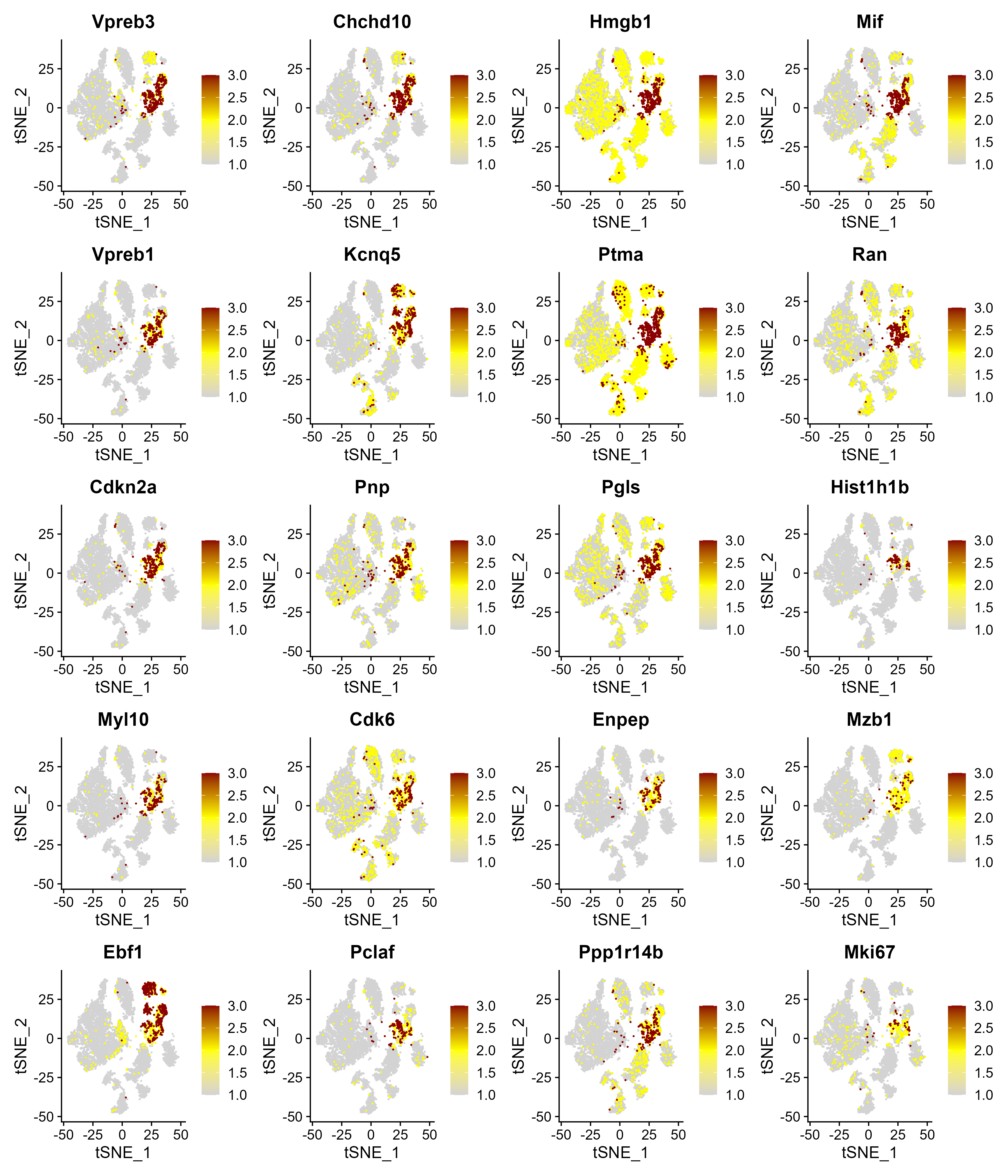

Supplement: Supplementary file 1 — Supplementary Material 1: Supplemental Fig. 1. Flow cytometry monitoring of the major immune cells in the BCRF8C and ZNF112 mouse models. Representative flow diagrams of PB samples from mice engrafted with BCRF8C (A) and ZNF112 (B) SCLL cells during leukemogenesis. Levels of CD4 + and CD8 + T-cells, Ly6C + CD11b + myeloid cells as well as CD19 + B-cells and CD49b + NK cells are shown from the PB, over 21 days (D7-D21) for BCRF8C and over 27 days (D14-D27) for ZNF112 cells. A progressive increase in Ly6C + CD11b + cells is seen in both models and all immune effector cells show a decrease. While the Ly6ChiCD11b + cell population (M-MDSC) shows only a modest increase, the proportion of Ly6CintD11b + PMN-MDSC show a highly significant increase. Leukemic cells are defined by the expression of GFP. The cell counts of each individual cell type included in this analysis are shown in (C) for BBC2 and (D) for ZNF112. [file 40164_2024_514_MOESM1_ESM.png]

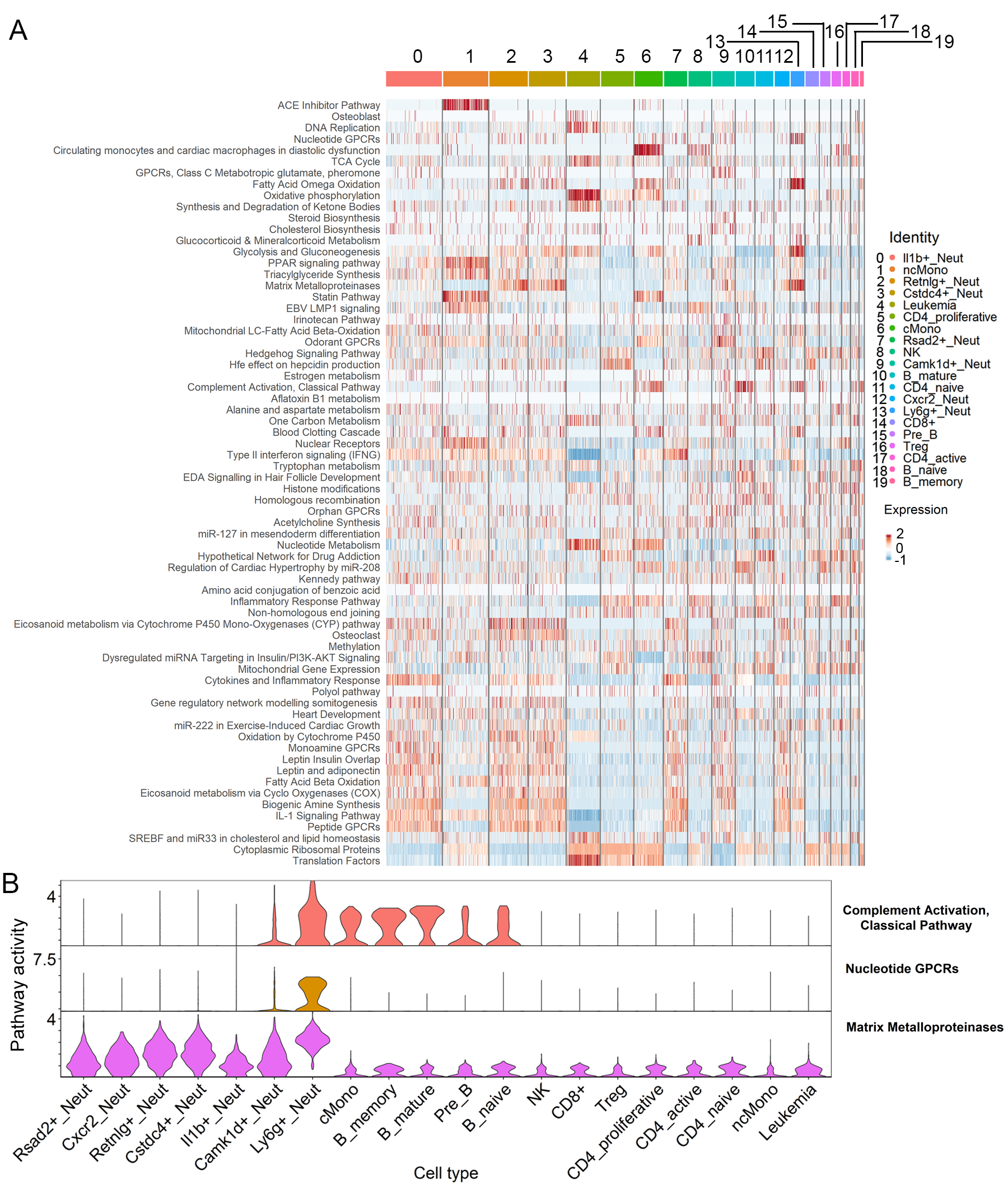

Supplement: Supplementary file 2 — Supplementary Material 2: Supplemental Fig. 2. Feature plots for gene defining B cell progenitor and cell proliferation in leukemia cells. Feature plots of genes highly expressed in the leukemia cell cluster show genes that are also expressed in pre-B cells (e.g. Vpreb3, Vpreb1, Ebf). In addition, genes relating to active cell cycling in proliferating leukemic cells including, Cdkn2a, Cdk6, Hist1a1b, and Mki67, are shown in the feature plots. [file 40164_2024_514_MOESM2_ESM.png]

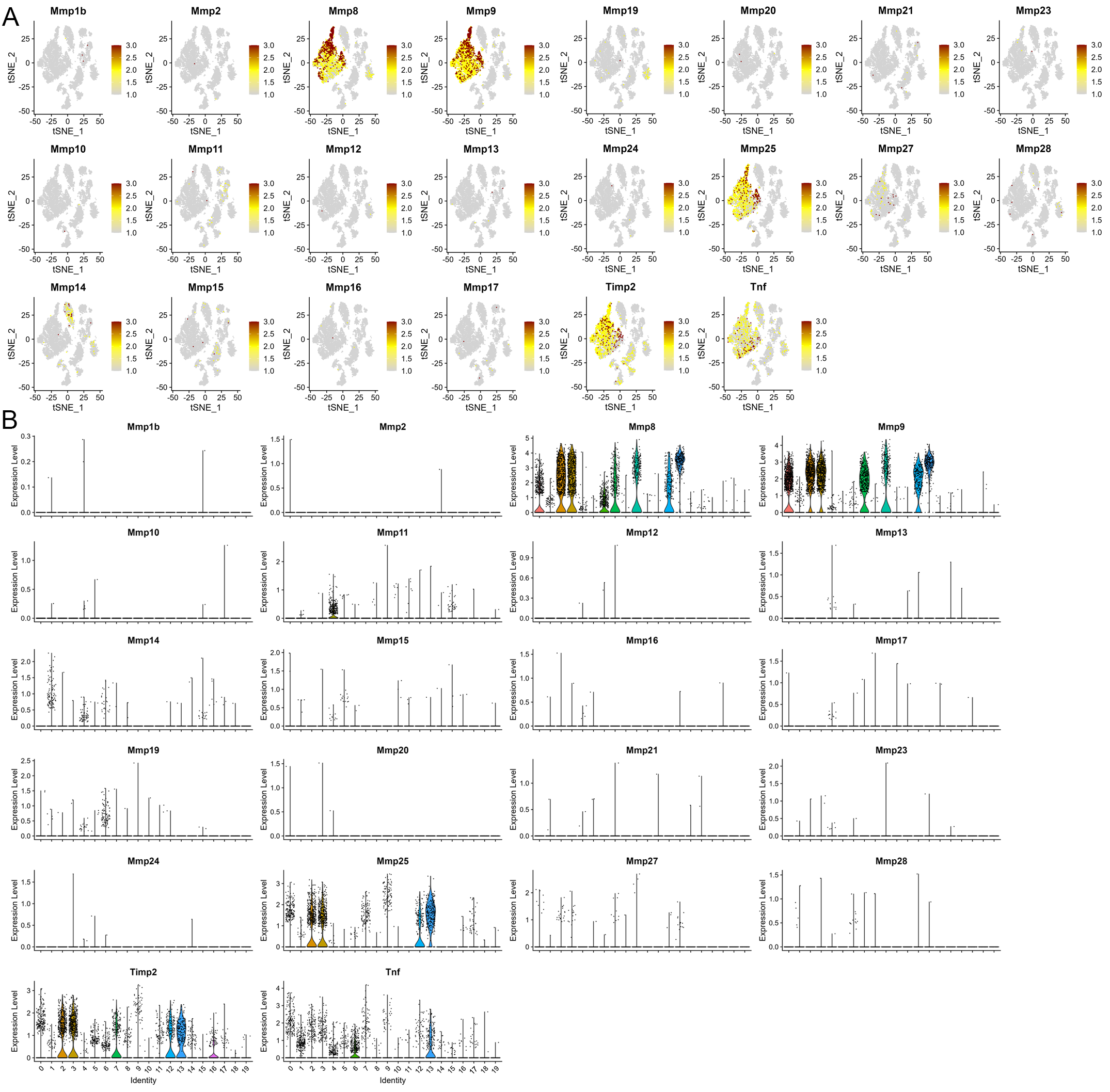

Supplement: Supplementary file 3 — Supplementary Material 3: Supplemental Fig. 3. Most significant wikipathways activated in different cell subtypes. Summary of most active pathways across the 20 clusters depicted in a heatmap in (A). Violin plots for selected pathways (B). [file 40164_2024_514_MOESM3_ESM.png]

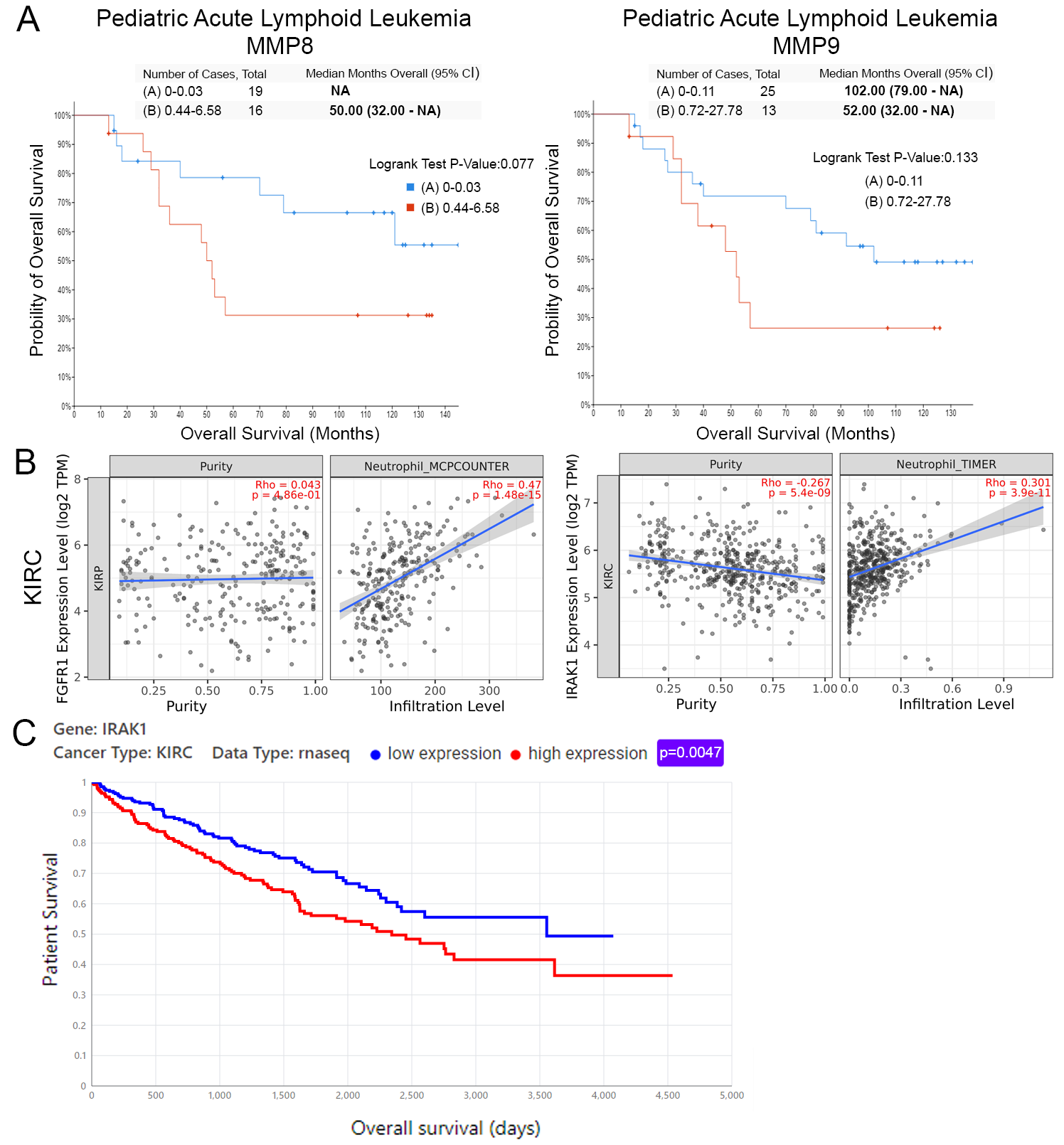

Supplement: Supplementary file 9 — Supplementary Material 9: Supplemental table 3 Markers identified in different neutrophil subclusters using FindMarker function. avg_logFC: log fold-change of the average expression between neutrophils in the target cluster and neutrophils from all the remaining clusters. Positive values indicate that the gene is more highly expressed in the target cluster. pct.1: The percentage of neutrophils where the gene is detected in the target cluster. pct.2: The percentage of neutrophils where the gene is detected in the remaining clusters. p_val_adj: Adjusted p-value, based on Bonferroni correction using all genes in the dataset. [file 40164_2024_514_MOESM9_ESM.png]

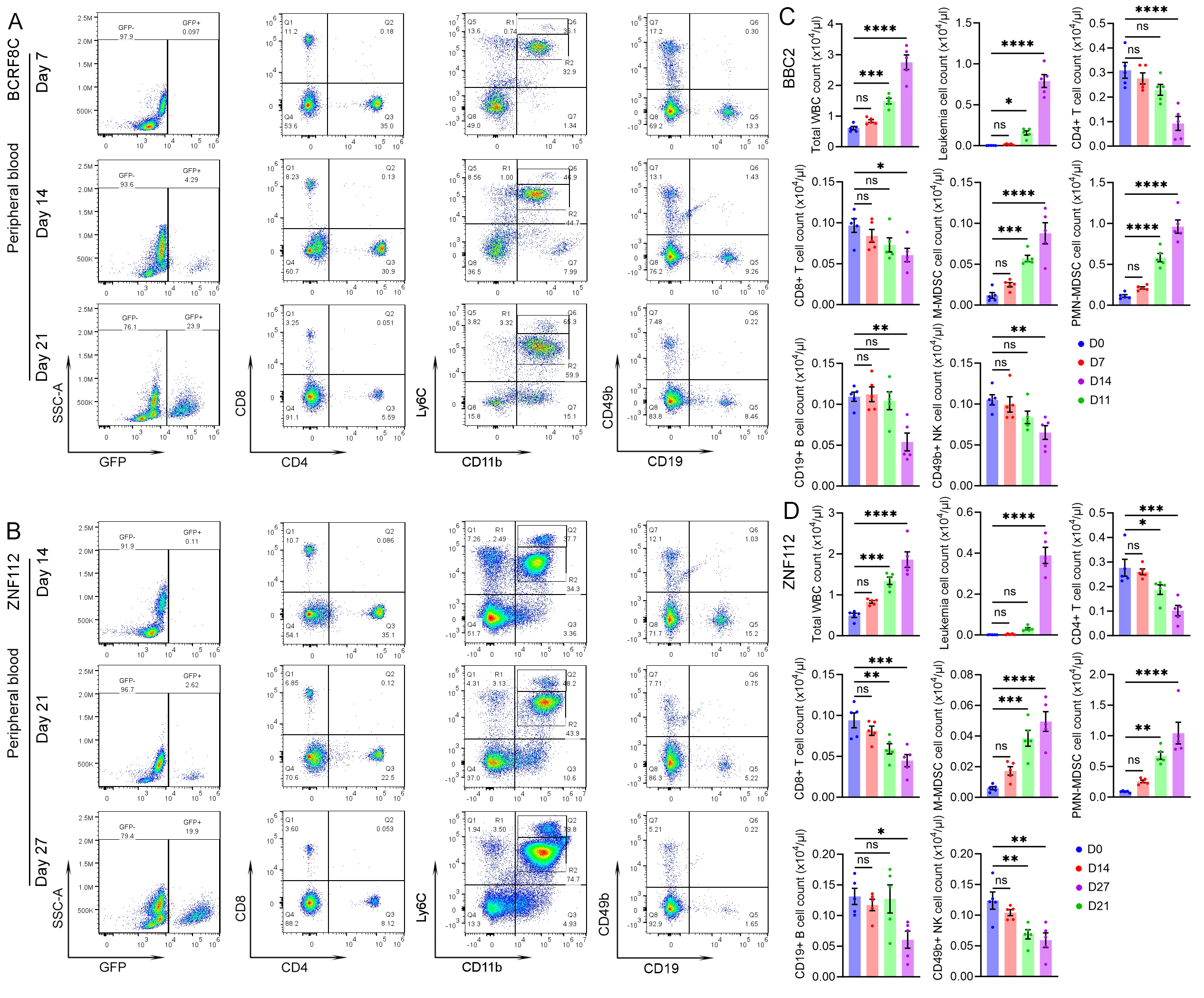

Supplement: Supplementary file 10 — Supplementary Material 10: Supplemental table 4 Modulated genes identified using the Monocle 3 workflow, whose expression is changing in a continuous manner over pseudotime. [file 40164_2024_514_MOESM10_ESM.png]

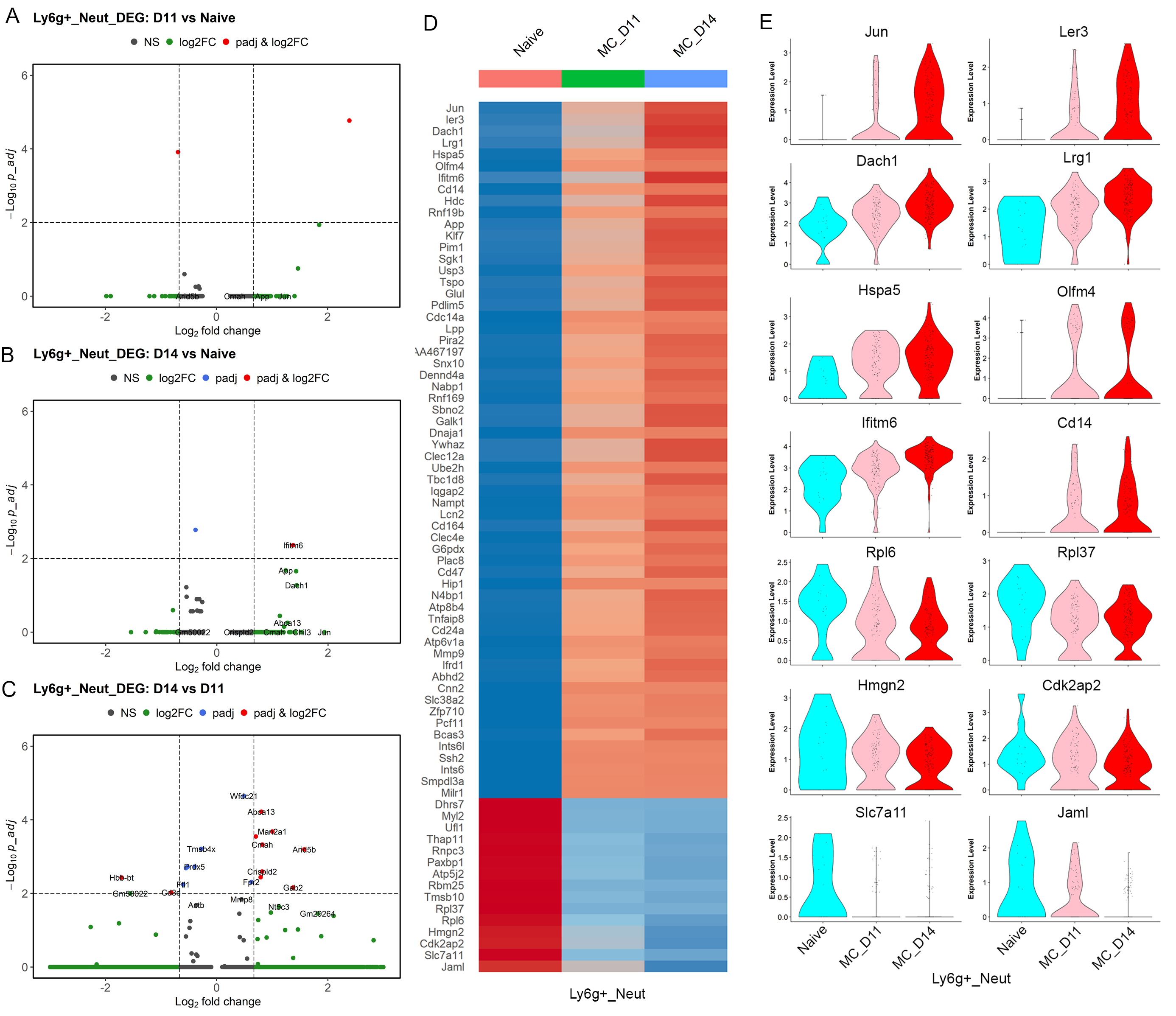

Supplement: Supplementary file 11 — Supplementary Material 11: Supplemental Table 5. Multivariate analysis of MMP8 and 9 in AML and KIRC using Cox proportional hazard ratio model for overall survival. From the multivariate survival analysis accounting for age, sex, and disease stage, the hazard ratio (HR) for MMP8 in AML is 1.465 in the high expression group compared to the low expression group with 95% CI [1.070, 2.005], which is statistically significant with p = 0.017; the HR for MMP8 in renal cell carcinoma is 1.556 in the high expression group compared to the low expression group with 95% confidence interval (CI) [1.136, 2.131], which is statistically significant with p = 0.00589; the HR for MMP9 in AML is 1.025 in the high expression group compared to the low expression group with 95% CI [0.756, 1.390], which is not statistically significant with p = 0.873; the HR for MMP9 in renal cell carcinoma is 1.365 in the high expression group compared to the low expression group with 95% CI [0.996, 1.870] with borderline significance p = 0.053. [file 40164_2024_514_MOESM11_ESM.png]
